# Psychosocial Well-Being at Work: Large-Sample Structural Validation of the Multidimensional Psychosocial Work Experience Scale for Employed Persons (MPWES)

**DOI:** 10.3390/ijerph23081080

**Published:** 2026-08-19

**Authors:** Evija Nagle, Iluta Skrūzkalne, Maksims Zolovs, Jeļena Perevozčikova, Otto Andersen, Andrejs Ivanovs, Ieva Reine

**Affiliations:** 1 Statistics Unit, Riga Stradiņš University, Dzirciema iela 16, LV-1007 Riga, Latviaieva.reine@rsu.lv (I.R.); 2 Institute of Life Sciences and Technology, Daugavpils University, Vienības iela 13, LV-5401 Daugavpils, Latvia; 3 School of Business and Law, University of Agder, Gimlemoen 25, 4630 Kristiansand, Norway; 4 Department of Public Health and Caring Sciences, Uppsala University, Husargatan 3, SE-751 22 Uppsala, Sweden

**Keywords:** psychosocial well-being, occupational health, employee well-being, working environment, job demands–resources model, confirmatory factor analysis, measurement invariance, scale validation, public health, psychosocial risk assessment

## Abstract

**Highlights:**

**Public health relevance—How does this work relate to a public health issue?**

- Psychosocial working conditions are modifiable determinants of employee health, work ability, sickness absence, and workforce sustainability.
- This study examined the structural validity of the Multidimensional Psychosocial Work Experience Scale for Employed Persons (MPWES) in a large heterogeneous sample of 1631 employed adults from four occupational sectors.

**Public health significance—Why is this work of significance to public health?**

- Confirmatory factor analysis supported a refined eight-factor structure of the MPWES, with acceptable model fit in both calibration and held-out validation subsample.
- The findings provide initial evidence of structural validity, reliability, discriminant validity, and measurement stability of the MPWES in the present sample.

**Public health implications—What are the key implications or messages for practitioners, policy makers and/or researchers in public health?**

- The MPWES may help identify a multidimensional psychosocial work environment profile relevant to employee well-being, including work intensity, autonomy, social inclusion, professional development, health risks, psychosomatic symptoms, and workplace harassment.
- Further studies using external validity criteria, longitudinal designs, and culturally diverse samples are needed before the scale can be considered fully validated for broader occupational or public health applications.

**Abstract:**

Psychosocial working conditions are important determinants of employee health, work ability, and organizational sustainability, yet existing instruments often assess job demands, job resources, psychosocial risks, or well-being outcomes separately. This study examined the structural validity of the Multidimensional Psychosocial Work Experience Scale for Employed Persons (MPWES), an instrument designed to assess a multidimensional occupational psychosocial profile comprising workplace conditions and demands, psychosocial resources, adverse exposures, health-related experiences, and subjective functioning. A cross-sectional validation study was conducted among 1631 employees from the pharmaceutical, energy, healthcare, and administrative sectors in Latvia. Confirmatory factor analysis with the robust WLSMV estimator was used to test the predefined measurement model. The refined eight-factor model demonstrated acceptable fit in the calibration subsample (N = 400; CFI = 0.930, TLI = 0.922, RMSEA = 0.066, SRMR = 0.078) and was replicated in the held-out validation subsample (N = 1231; CFI = 0.911, TLI = 0.901, RMSEA = 0.068, SRMR = 0.067). Reliability indices were acceptable for several domains, although some factors showed modest or borderline values. Discriminant validity was supported, with all HTMT values below 0.85, and measurement invariance testing supported configural, metric, scalar, and strict invariance across samples. The findings provide initial evidence of the MPWES’s structural validity and measurement stability in the present sample. However, further studies using external validity criteria, longitudinal designs, and culturally diverse samples are needed before the scale can be considered fully validated for broader occupational and public health applications.

## 1. Introduction

The concept of well-being occupies a central position in contemporary social science research, although no unified consensus exists regarding its precise definition, conceptual boundaries, or optimal measurement approaches [1,2,3]. Despite this heterogeneity, well-being is widely understood as a multidimensional construct that includes psychological, social, and contextual components [3]. Earlier assessments relied mainly on objective economic indicators, such as income, employment, and gross domestic product, but these indicators do not fully capture individuals’ lived experiences, perceived quality of life, or psychosocial functioning [4]. Consequently, contemporary research increasingly emphasizes subjective and psychosocial dimensions of well-being, including emotional functioning, relational quality, perceived security, and satisfaction with life and work [2,5].

In occupational settings, psychosocial well-being is shaped by the interaction between job demands, job resources, interpersonal dynamics, organizational structures, and perceived workplace safety. International frameworks developed by the World Health Organization (WHO) and the Organization for Economic Co-operation and Development (OECD) emphasize psychosocial working conditions and the quality of the working environment as important determinants of employee health and sustainable employment [6,7,8]. The Job Demands–Resources (JD-R) model further explains employee well-being through the balance between job demands, such as workload and time pressure, and job resources, such as autonomy, social support, and opportunities for development [9]. Together, these frameworks support the examination of workplace demands, resources, psychosocial risks, health-related experiences, and subjective functioning as related but conceptually distinct occupational domains.

Employee well-being is relevant not only for individual health but also for organizational effectiveness and workforce sustainability. Adverse psychosocial working conditions have been associated with job strain, burnout, moral distress, reduced productivity, absenteeism, and long-term disengagement, particularly in high-demand sectors such as healthcare [10,11,12,13,14]. Therefore, reliable assessment of occupational psychosocial conditions and employee-level experiences is important for occupational health research, organizational monitoring, prevention planning, and evidence-based workplace health promotion.

Although numerous instruments assess specific aspects of the psychosocial work context, such as job strain, burnout, job satisfaction, work characteristics, or psychosocial risk factors, many focus on selected dimensions rather than providing an integrated measurement structure for heterogeneous working populations. For example, some established instruments primarily assess psychosocial working conditions or organizational risk factors, such as the OECD working environment framework and the Job Demands–Resources Questionnaire [6,15], whereas broader approaches also consider employee well-being as an important workplace outcome [16,17,18]. These instruments are valuable, but they do not always integrate demand-related, resource-related, risk-related, symptom-related, and subjective functioning domains within a single structural measurement model [6,15,17,18].

The purpose of the present instrument is therefore broader than assessing the psychosocial work environment alone. It is intended to describe a multidimensional occupational psychosocial profile comprising workplace conditions and demands, psychosocial resources, adverse exposures, health-related experiences, and subjective functioning. Work Intensity, Health Risks, Autonomy, Social Inclusion, Professional Development, and Workplace Harassment primarily represent workplace conditions, resources, or exposures, whereas Psychosomatic Symptoms and Subjective Well-being represent employee-level experiences and functioning. These domains are conceptually related but are not treated as identical environmental characteristics or as interchangeable indicators of a single homogeneous construct.

The instrument was originally introduced as the Multidimensional Psychosocial Work Environment Scale for Employed Persons (MPWES), and its theoretical framework and preliminary psychometric evidence were established in a pilot study involving 213 employed adults. In the present study, the instrument’s name is refined to the Multidimensional Psychosocial Work Experience Scale for Employed Persons while retaining the established acronym MPWES. This refinement does not indicate the development of a new instrument or a change in its item content. Rather, it clarifies that the scale assesses a broader occupational psychosocial experience comprising workplace conditions and demands, psychosocial resources, adverse exposures, health-related experiences, and subjective functioning.

The present study represents the large-sample structural validation phase of the MPWES measurement model. Instead of redeveloping the instrument, it tests the initially specified factor structure, applies theoretically and statistically justified model refinement in a calibration subsample, and evaluates the refined structure in a held-out validation subsample derived from the same dataset.

Specifically, this study evaluates factorial validity, internal consistency, construct reliability, convergent and discriminant validity, and measurement invariance across the calibration and held-out validation subsamples. By examining the model’s structural validity and measurement stability, this study provides initial evidence of the MPWES’s suitability for assessing a multidimensional occupational psychosocial profile among employed adults. The present findings represent internal structural and psychometric evidence rather than external or comprehensive validation. Further studies using external validation criteria, longitudinal designs, independently recruited samples, and culturally diverse populations are needed to establish broader validation evidence.

### 1.1. Aim of the Study

This study aimed to examine the structural validity, reliability, convergent and discriminant validity, and internal split-sample stability of the previously developed Multidimensional Psychosocial Work Experience Scale for Employed Persons (MPWES) in a large heterogeneous sample of employed adults using confirmatory factor analysis.

### 1.2. Objectives of the Study

To achieve the overall aim, the following objectives were defined:

1. To test the initially specified factor structure of the MPWES in the calibration subsample using confirmatory factor analysis and to evaluate overall model fit.
2. To refine the measurement model where statistically and theoretically justified and to evaluate the refined factor structure without further modification in a held-out validation subsample.
3. To evaluate internal consistency and construct reliability using Cronbach’s alpha, McDonald’s omega, and Composite Reliability (CR), and to assess convergent validity using Average Variance Extracted (AVE).
4. To assess discriminant validity using the Heterotrait–Monotrait ratio of correlations (HTMT).
5. To examine measurement invariance across calibration and held-out validation subsample using multi-group CFA, including the evaluation of configural, metric, scalar, and strict invariance models.

## 2. Literature Review

### 2.1. Psychosocial Well-Being in the Work Context

Psychosocial well-being in the work context refers to employees’ subjective psychological and social functioning within their work environment [15,19]. It is shaped by, but conceptually distinct from, psychosocial working conditions, job demands, job resources, and psychosocial risks [6,15]. In the present study, the MPWES does not treat workplace conditions and employee-level experiences as identical constructs. Instead, it assesses a multidimensional psychosocial work experience profile comprising workplace conditions and demands, psychosocial resources, adverse exposures, health-related experiences, and subjective functioning. These domains are theoretically related but are modeled as conceptually distinct first-order dimensions. Unlike general or life-related well-being, psychosocial well-being is inherently context-specific, as it is closely linked to job demands, available resources, social relationships, organizational practices, and perceived workplace security [6,20]. Consequently, psychosocial well-being cannot be adequately understood or measured without explicit reference to the occupational environment in which it is embedded [21].

Empirical research in organizational and occupational health psychology consistently demonstrates that insufficient attention to psychosocial working conditions is associated with increased turnover intentions, absenteeism, and productivity losses [9,22]. These findings underscore the importance of clearly defining and accurately assessing psychosocial well-being as a central determinant of organizational sustainability.

Literature consistently conceptualizes psychosocial well-being at work as a multidimensional construct encompassing emotional, cognitive, and social components. These dimensions include perceived workload and work intensity, autonomy and control over work tasks, quality of interpersonal relationships, social support, opportunities for professional development, and perceptions of fairness and safety within the organization [3,15]. Meta-analytic evidence indicates that low autonomy and insufficient social support are among the strongest predictors of burnout and disengagement. In contrast, developmental opportunities and supportive organizational climates are positively associated with engagement and motivation [23]. Together, these components reflect employees’ capacity to cope with work-related demands, sustain motivation, and experience meaning and balance in their professional roles [21,22].

A key conceptual distinction in the study of employee well-being lies between context-free and domain-specific well-being, with psychosocial well-being at work representing a distinct and empirically relevant domain. Context-free well-being captures individuals’ overall life satisfaction and general emotional states, whereas psychosocial well-being focuses on experiences arising directly from employment conditions and organizational contexts [19,20]. This distinction is critical, as individuals may report high overall life satisfaction while simultaneously experiencing low psychosocial well-being at work due to excessive job demands, limited autonomy, or insufficient social support.

The Job Demands–Resources model provides a widely accepted theoretical framework for understanding psychosocial well-being in occupational settings. According to this model, psychosocial well-being results from the dynamic interplay between job demands, such as workload, emotional strain, and time pressure, and job resources, including autonomy, social support, feedback, and growth opportunities [9,15]. When job demands exceed available resources, the risk of emotional exhaustion, burnout, and disengagement increases. In contrast, adequate resources foster motivation, psychological resilience, and positive work functioning [22].

Beyond static associations, contemporary extensions of the JD-R model and Conservation of Resources theory [24] emphasize the emergence of gain spirals, whereby initial resource availability fosters engagement and performance, which in turn facilitate the acquisition of further resources over time [23,25]. Thus, psychosocial well-being reflects not merely the absence of negative outcomes but a dynamic balance between demands and resources embedded within organizational systems.

Importantly, psychosocial well-being extends beyond individual psychological states to encompass relational and organizational dimensions, including trust in management, social inclusion, a sense of belonging, and psychological safety. These factors play a crucial role in shaping employees’ emotional experiences, work engagement, and long-term sustainability in their roles, as well as influencing organizational performance and workforce stability [6,26]. These relational and organizational resources frequently operate in mutually reinforcing cycles, strengthening both individual well-being and organizational resilience. As such, psychosocial well-being serves as an important indicator of both individual functioning and organizational health.

In contemporary occupational research, psychosocial well-being is increasingly recognized as a central outcome linked to employee health, job performance, and organizational resilience. Higher levels of psychosocial well-being are associated with lower burnout risk, reduced absenteeism, and improved work effectiveness. In contrast, adverse psychosocial working conditions contribute to chronic stress, disengagement, and negative health outcomes [10,22,27,28]. These findings underscore the need for systematic, reliable, and context-sensitive measurement of psychosocial well-being, particularly in studies aimed at evaluating work environment quality and informing evidence-based organizational interventions.

### 2.2. Theoretical Foundations of the MPWES

This section adopts a comparative-integrative perspective to synthesize the principal theoretical frameworks that inform the conceptualization of psychosocial well-being. Rather than treating these frameworks as parallel explanatory models, the present study integrates their complementary contributions to establish a coherent foundation for multidimensional measurement.

Psychosocial well-being is conceptualized within theoretical traditions that examine how job demands, job resources, and organizational conditions shape employees’ psychological and social functioning [9,15]. The present framework draws upon three complementary perspectives: the Job Demands and Resources model, the World Health Organization’s conceptualization of psychosocial health at work, and the Organization for Economic Co-operation and Development’s framework for assessing the quality of the working environment.

The Job Demands and Resources model conceptualizes psychosocial well-being as a dynamic outcome emerging from the interaction between job demands and job resources [9]. Job demands refer to aspects of work that require sustained effort and entail psychological or physiological costs, including work intensity, emotional demands, and time pressure [15]. Job resources encompass factors that facilitate goal attainment, mitigate the adverse effects of job demands, and promote development, such as autonomy, social support, feedback, and growth opportunities [9]. Extensive empirical evidence demonstrates that an imbalance characterized by high demands and insufficient resources predicts psychological strain, emotional exhaustion, and reduced well-being. Conversely, adequate resources buffer adverse effects and foster engagement and adaptive functioning [9,15]. The Job Demands and Resources framework therefore provides the theoretical logic for distinguishing demand-related and resource-related domains within the MPWES measurement structure.

It is important to distinguish the measurement purpose of the MPWES from causal explanatory models of occupational well-being. In many occupational health frameworks, job demands, job resources, psychosocial risks, symptoms, and well-being are modeled as antecedents, mediators, or outcomes. MPWES does not assume that these domains are identical or causally ordered. Instead, it adopts a structural measurement perspective and assesses a multidimensional psychosocial work experience profile. Work Intensity and Health Risks represent demand- or risk-related workplace conditions; Autonomy, Social Inclusion, and Professional Development represent psychosocial resources; Workplace Harassment represents adverse interpersonal exposure; Psychosomatic Symptoms represent health-related experiences; and Subjective Well-being represents subjective functioning. These domains are conceptually related but are not treated as interchangeable indicators of a single homogeneous construct.

The World Health Organization perspective complements this structural model by situating psychosocial functioning and work-related health within a broader occupational health paradigm. World Health Organization guidelines conceptualize psychosocial health as dependent upon work organization, interpersonal relationships, social support, psychological safety, and institutional practices that sustain work ability and long-term functioning [8,29]. This perspective expands the analytical scope beyond task-level characteristics and incorporates organizational and systemic determinants of psychosocial functioning.

The Organization for Economic Co-operation and Development framework further extends this integrative model by providing a policy-oriented, internationally comparable framework for assessing the quality of the working environment [6]. The framework operationalizes working conditions across multiple dimensions, including work intensity, autonomy, safety, social relations, organizational clarity, employment security, and developmental opportunities [6,30]. Unlike models centered primarily on individual-level processes, the Organization for Economic Co-operation and Development perspective embeds psychosocial well-being within institutional and regulatory contexts, thereby facilitating cross-sector and cross-national comparability [31]. Several indicators within this framework align conceptually with job demands and job resources constructs [23,32], while others capture contextual features not explicitly addressed in traditional occupational stress models.

Integrating these three frameworks supports conceptualizing the MPWES as a multidimensional psychosocial work experience measurement model comprising demand-related, resource-related, risk-related, health-related, and subjective functioning domains. The Job Demands–Resources model provides the dynamic explanatory mechanism [23]. The World Health Organization perspective anchors psychosocial functioning and work-related health within occupational health [7,8]. The Organization for Economic Co-operation and Development framework introduces structural and policy-level comparability [30]. Together, they support a theoretically grounded measurement architecture capable of capturing both micro-level psychosocial processes and macro-level characteristics of the work environment.

In this study, psychosocial well-being is therefore not conceptualized solely as an outcome of work-related exposures. Rather, the MPWES operationalizes a multidimensional psychosocial work experience profile that includes workplace conditions and demands, perceived psychosocial resources, adverse exposures, health-related experiences, and subjective functioning. The purpose of the MPWES is to assess the configuration of these related but conceptually distinct occupational psychosocial domains, rather than to test directional causal pathways among demands, resources, symptoms, and well-being outcomes.

This reconceptualization differs from conventional applications of the Job Demands–Resources model, in which demands and resources are typically examined as antecedents of motivational, strain-related, or well-being outcomes. In contrast, the MPWES does not test causal pathways among demands, resources, symptoms, and well-being outcomes. Instead, it uses concepts derived from the JD-R, WHO, and OECD frameworks to define a structural measurement model of the psychosocial work environment. Therefore, the MPWES is best understood as a multidimensional psychosocial work experience scale rather than as a measure of the work environment alone, a pure well-being outcome scale, or a causal JD-R model.

Thus, the retained eight-factor structure integrates workplace conditions, psychosocial resources, adverse exposures, health-related experiences, and subjective functioning without treating them as environmental dimensions or interchangeable indicators of one homogeneous construct. Work Intensity reflects a job-demand domain, consistent with the Job Demands–Resources model and the occupational stress literature [15,23,32], whereas Health Risks captures risk-related exposure in the working environment, consistent with occupational health and working-environment frameworks [6,7,8]. Autonomy, Social Inclusion, and Professional Development represent psychosocial and developmental resources that correspond to job resource dimensions emphasized in JD-R theory and working environment quality frameworks [6,15,23,32]. Workplace Harassment reflects an adverse psychosocial risk domain, consistent with occupational health perspectives that emphasize psychosocial safety and harmful interpersonal work experiences [7,8]. Psychosomatic Symptoms represent work-related health experiences, whereas Subjective Well-being reflects employees’ perceived psychological functioning in relation to their broader work experience [2,5,21,33]. Thus, the retained eight-factor structure integrates demand-related, resource-related, risk-related, symptom-related, and subjective functioning domains without treating them as interchangeable indicators of one homogeneous construct.

Accordingly, the MPWES is conceptualized as a multidimensional profile composed of correlated first-order reflective domains [34,35]. The present study does not assume a single higher-order psychosocial well-being factor, nor does it treat the total construct as a homogeneous reflective latent variable [35,36]. Instead, each domain is interpreted as a distinct reflective dimension, and the overall instrument is intended to a multidimensional psychosocial work experience profile. This distinction is important for interpreting the CFA results, which evaluate the structure and differentiation of the first-order domains rather than testing a second-order or formative model [34,35,36,37].

Together, the JD-R, WHO, and OECD perspectives provide a coherent theoretical basis for distinguishing workplace demands, resources, risks, health-related experiences, and subjective functioning within the MPWES [6,7,21,24]. This integrated foundation informs the evaluation of the instrument as a multidimensional psychosocial work experience measurement model.

The subsequent section outlines the methodological approach for operationalizing these integrated theoretical constructs and evaluating the structural validity of the MPWES.

### 2.3. Measurement of Occupational Psychosocial Conditions, Experiences, and Well-Being

Researchers and practitioners rely on theoretically grounded and psychometrically robust instruments to assess psychosocial working conditions, occupational well-being, and work-related health outcomes. Empirical research in this field employs a wide range of self-report measures developed within distinct theoretical traditions and occupational contexts. To facilitate systematic comparison, the present review evaluates these instruments in terms of theoretical breadth, construct coverage, psychometric purpose, and practical applicability. Table 1 presents conceptually representative instruments that reflect dominant approaches to measuring psychosocial working conditions, employee well-being, engagement, burnout, and work-related health.

Established instruments differ in their measurement purposes and construct coverage. The Copenhagen Psychosocial Questionnaire (COPSOQ) and the HSE Management Standards Indicator Tool primarily assess psychosocial working conditions and organizational risks, whereas the WHO-5 Well-Being Index measures general subjective well-being, the Utrecht Work Engagement Scale (UWES) assesses work engagement, and burnout instruments focus on adverse occupational outcomes. The NIOSH Worker Well-Being Questionnaire provides a broader assessment that includes both work and non-work domains. These instruments are valuable but were developed for partly different purposes and do not necessarily integrate workplace conditions, psychosocial resources, adverse exposures, health-related experiences, and subjective functioning within a single structural measurement model. In contrast, the MPWES is intended to assess these related but conceptually distinct domains as components of a multidimensional psychosocial work experience profile.

Existing instruments differ substantially in their conceptual scope, intended purpose, and structural composition. Classic measures grounded in job design and occupational stress traditions focus primarily on job content, task characteristics, and strain processes, including task variety, autonomy, decision latitude, and effort–reward imbalance [38,39,40]. These models have provided foundational insights into the structural determinants of work-related stress. However, their emphasis on discrete job characteristics limits their capacity to capture the broader psychosocial experience of employees, particularly within increasingly complex and flexible work environments.

Subsequent measurement approaches expanded the focus to include effective and evaluative outcomes such as burnout, job satisfaction, and emotional well-being [16,33,41,42]. While these instruments have advanced the assessment of psychological functioning in occupational settings, they typically target specific outcome dimensions rather than modeling psychosocial well-being as a multidimensional construct that integrates contextual determinants and subjective experience.

More integrative approaches have emerged within the Job Demands and Resources framework, which explicitly models how demands and resources interact to shape employee well-being [15]. Complementary international frameworks, including those developed by the Organization for Economic Co-operation and Development and the National Institute for Occupational Safety and Health, incorporate broader psychosocial, organizational, and contextual indicators [6,17]. These frameworks expand conceptual coverage by integrating structural and environmental determinants of well-being. Nevertheless, many such tools are designed primarily for organizational monitoring, surveillance, or policy evaluation rather than as unified psychometric instruments for individual-level validation of latent constructs.

Compared with these established instruments, the MPWES was developed to assess a multidimensional psychosocial work experience profile. Its purpose is not to replace existing measures but to complement them by integrating workplace demands and conditions, psychosocial resources, adverse exposures, psychosomatic symptoms, and subjective functioning within a single structural measurement framework. Although the scale was developed for use in heterogeneous employed populations, the present study does not establish the validity of direct comparisons between occupational sectors because sector-level measurement invariance was not examined.

Collectively, the literature shows that existing instruments provide valuable but partly distinct perspectives on psychosocial work and employee well-being. Some instruments emphasize job characteristics or psychosocial risks; others focus on engagement, burnout, general well-being, or broader worker well-being. However, fewer instruments are designed to model demand-related, resource-related, risk-related, symptom-related, and subjective functioning domains simultaneously within one structural measurement framework for heterogeneous employed populations. This gap supports the need for large-sample structural validation of the MPWES as an integrated psychosocial work environment measurement model.

**Table 1:** Representative instruments and frameworks for assessing occupational psychosocial conditions, employee experiences, well-being, and work-related outcomes.

Reference	Name	Goal	Factors	Primary Focus Relative to the MPWES
[38]	JDS	Assess work design effects on motivation and satisfaction	Work variety, task identity, task importance, autonomy, feedback	Job characteristics only
[39]	JCQ	Assess job demands, control, and social support	Work requirements, freedom of decision, social support	Demands/control/support only
[16]	MBI	Assess occupational burnout	Emotional exhaustion, depersonalization, and personal accomplishment.	Burnout outcome only
[41]	JSS	Assess job satisfaction	Remuneration, career development, management support, benefits, colleagues, communication	Job satisfaction only
[43]	Ryff PWB Scales	Assess psychological well-being	Autonomy, environmental management, personal growth, positive relationships, life purpose, self-acceptance	General well-being only
[40]	ERI	Assess effort–reward imbalance	Effort, reward, overcommitment	Effort–reward imbalance only
[42]	JAWS	Assess work-related emotions	Joy, Enthusiasm, Pride, Satisfaction, Comfort, Anger, Frustration, Anxiety, Depression, Disappointment	Work emotions only
[15]	JD-R	Assess job demands and resources	Scope of work physical demands, cognitive requirements, emotional requirements, physical. Requirements, organizational resources, personal resources, and environmental resources	Demands/ resources only
[6]	OECD Working Environment Guidelines	Assess working environment quality	Physical security, work intensity, social relations, job autonomy, development opportunities	Policy indicators only
[17]	NIOSH WellBQ	Assess overall worker well-being	Working environment, life outside work, health, social support, and financial security	Broad worker well-being

JDS = Job Diagnostic Survey; JCQ = Job Content Questionnaire; MBI = Maslach Burnout Inventory; JSS = Job Satisfaction Survey; PWB = Psychological Well-Being; ERI = Effort–Reward Imbalance Questionnaire; JAWS = Job-Related Affective Well-Being Scale; JD-R = Job Demands–Resources; NIOSH WellBQ = National Institute for Occupational Safety and Health Worker Well-Being Questionnaire; MPWES = Multidimensional Psychosocial Work Experience Scale for Employed Persons.

Table 1 shows that existing instruments provide valuable but distinct measurement perspectives. Job design and occupational stress instruments, such as the Job Diagnostic Survey, Job Content Questionnaire, and Effort–Reward Imbalance Questionnaire, focus primarily on specific job characteristics, demands, control, or reward imbalance. Outcome-oriented instruments, such as the Maslach Burnout Inventory, Job Satisfaction Survey, Ryff Psychological Well-Being Scales, and Job-Related Affective Well-Being Scale, assess specific psychological, affective, motivational, or adverse occupational outcomes. Broader frameworks, such as the OECD working environment framework and NIOSH WellBQ, provide comprehensive indicators of work environment quality or worker well-being, but are not designed specifically to test the MPWES structural configuration. In contrast, the MPWES integrates demand-related, resource-related, risk-related, symptom-related, and subjective functioning domains within one structural measurement model for heterogeneous employed populations. Therefore, the contribution of the MPWES lies not in replacing established validated instruments, but in offering an integrated measurement framework for assessing workplace conditions and demands, psychosocial resources, adverse exposures, health-related experiences, and subjective functioning within a multidimensional psychosocial work experience profile.

Overall, existing instruments provide valuable but partly distinct perspectives on psychosocial working conditions, employee well-being, engagement, burnout, and broader worker functioning. The MPWES complements these approaches by integrating workplace demands and conditions, psychosocial resources, adverse exposures, health-related experiences, and subjective functioning within a multidimensional psychosocial work experience profile. Its intended applicability to heterogeneous employed populations creates potential for future cross-sectoral research; however, direct comparisons between occupational sectors require dedicated measurement invariance testing. These considerations support the need for large-sample structural evaluation of the MPWES while avoiding claims of comprehensive or cross-sectoral validation.

## 3. Methods

### 3.1. Participants and Procedure

A cross-sectional, two-stage internal split-sample psychometric evaluation design was employed to examine the structural validity and measurement stability of the Multidimensional Psychosocial Work Experience Scale for Employed Persons (MPWES). In the first stage, a calibration subsample (N = 400) was used to test the initially specified nine-factor measurement model and to refine the model where statistically and theoretically justified. In the second stage, a held-out validation subsample (N = 1231) was used to evaluate the refined measurement model without further modification. Thus, the analytical strategy consisted of initial model testing, model refinement, and internal split-sample validation rather than external replication or strict confirmation of the original model.

The total analytic sample was randomly divided into two non-overlapping subsamples before model refinement. The calibration subsample included 400 participants, while the remaining 1231 participants formed the held-out validation subsample. The held-out validation subsample therefore represents an internal split-sample validation rather than validation in an externally recruited independent sample. The split was not stratified by demographic or occupational characteristics. However, post hoc comparisons showed no statistically significant differences between the calibration and held-out validation subsample in gender, age, length of service, or employment sector, supporting the comparability of the two subsamples (Table 2).

Participants were recruited using convenience sampling from four organizations in Latvia, with one organization representing each of the following employment sectors: pharmaceutical manufacturing, energy services, healthcare, and administrative and support services. Approximately 2000 employed individuals were invited to participate, and 1631 submitted questionnaires that met the study’s eligibility and data-quality criteria. Because the number of invitations was approximate, a precise response rate could not be calculated. Eligibility criteria required participants to be at least 18 years old, currently employed full-time or part-time, and sufficiently proficient in Latvian to understand the questionnaire items. Participants were recruited from four organizations, with one organization representing each of the four employment sectors: pharmaceutical manufacturing, energy services, healthcare, and administrative and support services. Consequently, sector membership was fully confounded with organization membership, and the effects of employment sector could not be distinguished from organization-specific characteristics.

The total sample comprised 1631 participants, including 1053 women, 575 men, and three participants with missing gender information. The mean age was 45.86 years (SD = 11.90), and the mean length of service was 13.47 years (SD = 11.83). Participants represented the energy, pharmaceutical, healthcare, and administrative and support service sectors, with 78 cases classified as other or missing sector information.

The calibration subsample included 400 participants, comprising 260 women, 139 men, and one participant with missing gender information. The mean age was 45.72 years (SD = 11.84), and the mean length of service was 13.35 years (SD = 11.78). The sample included 120 participants from the energy sector, 109 from pharmaceuticals, 84 from healthcare, 68 from administrative and support services, and 19 participants with other or missing sector information.

The held-out validation subsample included 1231 participants, comprising 793 women, 436 men, and two participants with missing gender information. The mean age was 45.91 years (SD = 11.92), and the mean length of service was 13.51 years (SD = 11.85). The sample included 369 participants from the energy sector, 333 from pharmaceuticals, 257 from healthcare, 213 from administrative and support services, and 59 with other or missing sector information.

Overall, no statistically significant differences were observed between the calibration and held-out validation subsample in gender, age, length of service, or employment sector, supporting their comparability for the cross-validation analyses (Table 2).

Data collection was conducted between 10 September and 20 November 2024 using a self-administered online questionnaire. Personalized email invitations were distributed in collaboration with participating organizations, and reminder emails were sent at two-week intervals to enhance participation. Before completing the questionnaire, participants received detailed information about the study objectives and procedures and provided electronic informed consent. Participation was voluntary, anonymity was ensured, and participants were informed of their right to withdraw at any time without penalty.

The study protocol was approved by the Ethics Committee of Riga Stradiņš University (Decision No. 2-PEK-4/495/2024) and was conducted in accordance with the principles of the Declaration of Helsinki.

### 3.2. Measurement Instrument

The multidimensional occupational psychosocial profile was assessed using the Multidimensional Psychosocial Work Experience Scale for Employed Persons (MPWES), a self-report instrument developed and pilot-tested in a previous study.

The instrument was originally introduced as the Multidimensional Psychosocial Work Experience Scale for Employed Persons (MPWES). In the present study, the wording of the name was refined while retaining the established acronym MPWES. This refinement does not indicate the development of a new instrument or a change in its item content; rather, it clarifies that the scale includes workplace conditions and demands, psychosocial resources, adverse exposures, health-related experiences, and subjective functioning. The present study evaluates the previously specified measurement model in a large sample of employed adults.

Based on the pilot-study findings, the Financial Safety factor was excluded before the present large-sample structural evaluation because it demonstrated insufficient psychometric performance and limited stability as a distinct reflective latent domain. Although financial security is relevant to broader worker well-being frameworks, the pilot findings did not provide sufficiently stable evidence that the included items formed a coherent and distinct component of the MPWES measurement structure. The refined MPWES should therefore be interpreted as assessing selected dimensions of a multidimensional occupational psychosocial profile rather than as a comprehensive measure of all possible occupational, financial, or personal determinants of employee well-being.

Accordingly, the present study initially tested an a priori nine-factor measurement model comprising 43 items.

The model comprised the following psychosocial dimensions: Subjective well-being, Inclusion, Social support, Workplace harassment, Work intensity, Psychosomatic symptoms, Professional development, Health risks, and Autonomy.

The initial measurement model retained the item allocation established during the pilot phase and was subsequently subjected to confirmatory factor analysis. The development of the MPWES was theoretically informed by the World Health Organization’s approach to psychological well-being, the Organization for Economic Co-operation and Development framework for assessing the quality of the working environment, and the Job Demands–Resources model, which together guided the operationalization of job demands, job resources, and employees’ subjective experiences of psychosocial well-being in the work context.

All items were administered using structured response formats, primarily six-point Likert-type scales assessing agreement or frequency, with selected items employing dichotomous response options to capture the presence or absence of specific work-related conditions. Following reverse coding where applicable, higher domain scores indicate a more favorable status on the corresponding dimension, such as greater subjective well-being, stronger psychosocial resources, lower exposure to adverse working conditions, or fewer health-related symptoms. The full list of final MPWES items, response formats, and scoring procedures is provided in the Supplementary Materials. Following the removal of V5 and V11 during model refinement, the retained items were renumbered consecutively from 1 to 41 in the final questionnaire. To preserve correspondence with the original analytical dataset, the CFA tables retain the original item codes (V1–V43).

To improve transparency regarding the final version of the instrument, Table 3 summarizes the number of items per factor, example item content, response format, scoring direction, and theoretical source for each MPWES domain.

Supplementary Material S1 presents the English version of the instrument, and Supplementary Material S2 presents the Latvian version used in the present study.

### 3.3. Statistical Analysis

Data preparation and statistical analyses were conducted using SPSS version 29.0 (IBM Corp., Armonk, NY, USA) and the R statistical environment (R Core Team, 2023, Vienna, Austria). Descriptive statistics were computed to characterize the sample’s demographic profile and to examine the distributional properties of item responses. Before analysis, the data were screened for completeness and consistency.

The proportion of missing data was below 2% across indicators. Missingness was examined descriptively across items, and no systematic concentration of missing responses was observed. Given the very low proportion of missing data, pairwise deletion was applied to retain available information for the CFA based on polychoric correlations [44]. However, because pairwise deletion can potentially affect covariance structures, particularly in CFA models with ordinal or dichotomous indicators, the results should be interpreted with this limitation in mind. Future analyses should consider sensitivity checks using listwise deletion or other missing-data handling approaches appropriate for ordinal CFA. Organization-level clustering was not incorporated into the CFA models because only four organizations were included, with each organization representing a different employment sector. This structure did not permit reliable separation of organization-level and sector-level effects or the use of multilevel CFA or cluster-robust estimation. Individual respondents were therefore treated as the unit of analysis.

The structural properties of the Multidimensional Psychosocial Work Experience Scale for Employed Persons (MPWES) were evaluated using confirmatory factor analysis (CFA) [34]. The CFA models were specified as correlated first-order factor models, with each MPWES domain treated as a distinct reflective latent factor indicated by its corresponding items. A single higher-order factor and a formative composite model were not tested because the instrument was conceptualized as a multidimensional psychosocial work experience profile comprising conceptually distinct domains rather than as a single homogeneous latent construct.

Given the ordinal and dichotomous nature of the MPWES items, the Robust Weighted Least Squares Mean and Variance adjusted (WLSMV) estimator was employed [45]. All six-point Likert-type items and dichotomous yes/no items were treated as categorical indicators. Thresholds were estimated for each item category, and the measurement model was estimated using the appropriate polychoric correlation matrix, with tetrachoric correlations represented as the special case for dichotomous indicators. This approach allowed ordinal and dichotomous indicators to be modeled jointly within the same CFA framework. The WLSMV estimator was selected because it is appropriate for ordered categorical indicators and does not assume multivariate normality. In each model, the first-order latent factors were allowed to correlate freely. Latent factor correlations were estimated as part of the confirmatory factor analysis using the WLSMV estimator and were obtained from the standardized latent covariance matrix.

Model fit was assessed using the Comparative Fit Index (CFI), Tucker–Lewis Index (TLI), Root Mean Square Error of Approximation (RMSEA), and Standardized Root Mean Square Residual (SRMR). Thresholds for acceptable fit were defined as CFI and TLI ≥ 0.90, RMSEA < 0.08, and SRMR < 0.08 [35,46].

The analysis proceeded in three sequential steps. First, the predefined nine-factor model was tested in the calibration subsample without modifications. Second, model refinement was conducted after evaluating global model fit, standardized factor loadings, conceptual overlap between factors, and the substantive meaning of potential modifications. Third, the refined model was cross-validated in the held-out validation subsample without further modification. This approach distinguished the initial model evaluation from subsequent refinement and cross-validation.

Following evaluation of the initial nine-factor model, an iterative refinement process was conducted. During the calibration-stage refinement, V5 and V11 were removed because their standardized factor loadings were below 0.50 and their substantive contribution to the intended factors was considered limited. The 0.50 value was used as a calibration-stage screening criterion together with theoretical evaluation rather than as an automatic item-deletion rule. No additional items were removed on the basis of the results obtained in the held-out validation subsample. Conceptually overlapping factors, namely Inclusion and Social Support, were merged into a single Social Inclusion domain because both represented relational aspects of the psychosocial work environment related to belonging, acceptance, interpersonal integration, and perceived support at work. Although inclusion and social support can be distinguished conceptually, the pilot and calibration analyses indicated substantial empirical proximity between these domains. Therefore, the combined Social Inclusion factor was interpreted as a broader relational resource domain rather than as a purely statistical solution.

To account for shared residual variance between semantically related items within the same conceptual domains, three within-factor residual covariances were specified between V27–V28, V18–V3, and V14–V15. All model refinements were guided by both statistical criteria (standardized factor loadings and theoretically interpretable modification indices) and substantive theoretical considerations. Residual covariances were specified only between semantically related items within the same conceptual domains and were used cautiously to account for local item similarity rather than to mask broader model misspecification or item redundancy. No additional modifications were introduced in the held-out validation subsample. The modification indices and expected parameter changes for the three residual covariances retained during model refinement are presented in Supplementary Material Table S2. The unstandardized residual covariance estimates, standard errors, and statistical significance levels were examined in both the calibration and held-out validation subsamples. In addition, because V27 and V28 showed comparatively weak standardized loadings in the held-out validation subsample, a sensitivity CFA excluding these two indicators was estimated to examine whether the remaining measurement model retained acceptable global fit. The sensitivity model was compared with the retained-item model using CFI, TLI, RMSEA, and SRMR.

To ensure transparency in the model refinement process, each modification was evaluated using both statistical criteria and theoretical justification. The refinement decisions are summarized in Table 4.

Internal consistency was assessed using Cronbach’s alpha [47], McDonald’s omega [48], and Composite Reliability (CR) [37]. Convergent validity was evaluated using Average Variance Extracted (AVE) [37], whereas discriminant validity was examined using the Heterotrait–Monotrait (HTMT) ratio of correlations, applying a strict cutoff value of <0.85 [49].

Measurement invariance across the calibration and held-out validation subsamples was examined using multi-group CFA with the WLSMV estimator. Configural, metric, scalar, and strict invariance models were tested sequentially. Configural invariance assessed whether the same factor structure was supported in both subsamples; metric invariance constrained factor loadings to equality; scalar invariance additionally constrained item thresholds; and strict invariance further constrained residual variances. Invariance was evaluated by examining changes in CFI, RMSEA, and SRMR between successively constrained models, with invariance supported when no meaningful deterioration in model fit was observed. Because the two groups were randomly generated subsamples of the same dataset, these analyses were interpreted as evidence of internal split-sample measurement stability rather than invariance across occupational sectors, independently recruited populations, or cultural groups.

All statistical tests were conducted using a two-tailed significance level of *p* < 0.05.

Large language models were used exclusively for language editing and stylistic refinement of the manuscript. No artificial intelligence tools were used for data analysis, interpretation of results, or generation of scientific content. All authors take full responsibility for the integrity and accuracy of the work.

## 4. Results

### 4.1. Confirmatory Factor Analysis and Model Fit in the Calibration Subsample

Confirmatory factor analysis (CFA) was first conducted in the calibration subsample (N = 400) to evaluate the predefined nine-factor measurement model before any model modifications were introduced. The initial nine-factor model demonstrated suboptimal fit to the data: CFI = 0.877, TLI = 0.879, RMSEA = 0.089, and SRMR = 0.080. The CFI and TLI values were below 0.90, RMSEA exceeded the prespecified threshold of 0.08, and SRMR was at the conventional upper boundary, indicating that model refinement was warranted. These results were therefore used as the baseline model fit before refinement.

Examination of standardized factor loadings, conceptual overlap between factors, and theoretically interpretable modification indices guided the refinement process. Specifically, items V5 and V11 were removed because their standardized factor loadings were below 0.50. The Inclusion and Social Support factors were merged into a single Social Inclusion domain because they demonstrated both conceptual overlap and empirical proximity, reflecting related relational and supportive aspects of the psychosocial work environment. In addition, three residual covariances were added between semantically related items within the same conceptual domains. The refined eight-factor model was then estimated in the calibration subsample. The model demonstrated acceptable global fit: CFI = 0.930, TLI = 0.922, RMSEA = 0.066, and SRMR = 0.078, indicating that the proposed structure adequately reproduced the observed covariance matrix.

All freely estimated factor loadings were statistically significant (*p* < 0.001). In the calibration subsample, standardized factor loadings ranged from 0.540 to 0.997. At the factor level, loadings ranged from 0.624 to 0.855 for Subjective Well-being, from 0.540 to 0.724 for Social Inclusion, from 0.676 to 0.975 for Workplace Harassment, from 0.588 to 0.920 for Work Intensity, from 0.863 to 0.889 for Psychosomatic Symptoms, from 0.579 to 0.921 for Professional Development, from 0.879 to 0.997 for Health Risks, and from 0.841 to 0.865 for Autonomy. Overall, most items demonstrated moderate-to-strong relationships with their respective latent factors. Detailed item-level results, including unstandardized estimates, standard errors, *p*-values, standardized factor loadings, and modification indices, are presented in Supplementary Material S3, Tables S1 and S3.

Latent factor correlations in the calibration subsample ranged from −0.678 to 0.802. The strongest positive correlation was observed between Subjective Well-being and Social Inclusion (r = 0.802), whereas the strongest negative correlation was found between Social Inclusion and Work Intensity (r = −0.678). Other comparatively strong associations were observed between Subjective Well-being and Work Intensity (r = −0.649), Subjective Well-being and Psychosomatic Symptoms (r = 0.635), and Work Intensity and Psychosomatic Symptoms (r = −0.618). None of the absolute correlation coefficients exceeded 0.85, suggesting that the eight latent domains were related but did not demonstrate excessive overlap. This pattern was consistent with the specification of a correlated first-order factor model. The complete latent factor correlation matrix for the calibration subsample is presented in Table 5.

More detailed results, including standard errors, z-statistics, *p*-values, and 95% confidence intervals for each latent factor correlation, are provided in Supplementary Material Table S5.

### 4.2. Cross-Validation in the Held-Out Validation Subsample

The refined eight-factor model was subsequently evaluated in the held-out validation subsample (N = 1231) without introducing any additional model modifications. The model demonstrated acceptable global fit: CFI = 0.911, TLI = 0.901, RMSEA = 0.068, and SRMR = 0.067. These findings supported the overall structural fit of the refined model in the held-out validation subsample; however, they did not indicate complete item-level replication because two Work Intensity indicators demonstrated comparatively weak factor loadings.

All freely estimated factor loadings were statistically significant (*p* < 0.001). In the held-out validation subsample, standardized factor loadings ranged from 0.385 to 0.955. At the factor level, loadings ranged from 0.617 to 0.876 for Subjective Well-being, from 0.530 to 0.738 for Social Inclusion, from 0.740 to 0.870 for Workplace Harassment, from 0.385 to 0.954 for Work Intensity, from 0.737 to 0.886 for Psychosomatic Symptoms, from 0.679 to 0.955 for Professional Development, from 0.788 to 0.948 for Health Risks, and from 0.781 to 0.873 for Autonomy. Overall, most items demonstrated moderate-to-strong relationships with their respective latent factors. However, two Work Intensity indicators, V27 (λ = 0.407) and V28 (λ = 0.385), showed standardized loadings below 0.50 and should therefore be interpreted cautiously. These findings indicate that, although acceptable global model fit was obtained, the item-level structure of the Work Intensity factor was not fully replicated in the held-out validation subsample. The comparatively weak loadings of V27 and V28 also raise the possibility of item instability and local dependence, particularly because residual covariance between these indicators was specified during calibration-stage model refinement. Detailed item-level results, including unstandardized estimates, standard errors, *p*-values, and standardized factor loadings, are presented in Supplementary Material S3 Table S4.

Latent factor correlations in the held-out validation subsample ranged from −0.644 to 0.684. The strongest positive correlation was observed between Subjective Well-being and Social Inclusion (r = 0.684), whereas the strongest negative correlation was found between Workplace Harassment and Work Intensity (r = −0.644). Other comparatively strong associations were observed between Subjective Well-being and Psychosomatic Symptoms (r = 0.651), Work Intensity and Psychosomatic Symptoms (r = −0.610), and Workplace Harassment and Psychosomatic Symptoms (r = 0.577). None of the absolute correlation coefficients exceeded 0.85, indicating that the latent factors were related without demonstrating excessive overlap. The latent factor correlation pattern was broadly comparable with that observed in the calibration subsample, although the weak loadings of V27 and V28 indicated instability at the item level within the Work Intensity factor. The complete latent factor correlation matrix for the held-out validation subsample is presented in Table 6.

### 4.3. Residual Covariances and Sensitivity Analysis of the Work Intensity Factor

The residual covariance estimates for the three item pairs introduced during calibration-stage model refinement were examined in both subsamples. In the calibration subsample, all three residual covariances were statistically significant: V27–V28, estimate = 0.501 (SE = 0.041, *p* < 0.001; standardized estimate = 0.773); V18–V3, estimate = 0.370 (SE = 0.034, *p* < 0.001; standardized estimate = 0.612); and V14–V15, estimate = 0.315 (SE = 0.029, *p* < 0.001; standardized estimate = 0.501). In the held-out validation subsample, the corresponding residual covariances also remained statistically significant: V27–V28, estimate = 0.597 (SE = 0.020, *p* < 0.001; standardized estimate = 0.706); V18–V3, estimate = 0.342 (SE = 0.021, *p* < 0.001; standardized estimate = 0.610); and V14–V15, estimate = 0.248 (SE = 0.016, *p* < 0.001; standardized estimate = 0.419). Overall, the residual associations were broadly stable across the two subsamples. However, the comparatively strong standardized residual covariance between V27 and V28 in both subsamples indicated persistent local dependence between these indicators (see Table 7).

Because V27 and V28 showed standardized factor loadings below 0.50 in the held-out validation subsample, a sensitivity CFA excluding both indicators was conducted in both subsamples. In the calibration subsample, the sensitivity model yielded χ^2^WLSMV(672) = 1765.611, *p* < 0.001, CFI = 0.929, TLI = 0.922, RMSEA = 0.064, and SRMR = 0.086. Compared with the retained-item model (CFI = 0.930, TLI = 0.922, RMSEA = 0.066, and SRMR = 0.078), the sensitivity model showed a slightly lower RMSEA but a higher SRMR that exceeded the prespecified threshold of 0.08.

In the held-out validation subsample, the sensitivity model yielded χ^2^WLSMV(672) = 4480.409, *p* < 0.001, CFI = 0.907, TLI = 0.897, RMSEA = 0.068, and SRMR = 0.072. Compared with the retained-item model (CFI = 0.911, TLI = 0.901, RMSEA = 0.068, and SRMR = 0.067), removal of V27 and V28 resulted in slightly lower CFI and TLI values and a higher SRMR, while RMSEA remained unchanged. Thus, excluding V27 and V28 did not produce a consistently improved measurement model. Because their removal also reduced the Work Intensity factor to two indicators, the sensitivity model should be interpreted cautiously. Overall, the broader eight-factor configuration retained broadly similar global fit, but the results confirmed item-level instability and local dependence within the Work Intensity factor.

### 4.4. Internal Consistency and Convergent Validity Across Samples

Internal consistency, construct reliability, and convergent validity were evaluated separately in the calibration subsample (N = 400) and the held-out validation subsample (N = 1231). Cronbach’s alpha, McDonald’s omega, composite reliability (CR), and average variance extracted (AVE) were calculated for each of the eight MPWES factors. The corresponding results are presented in Table 8.

In the calibration subsample, Cronbach’s alpha values ranged from 0.59 to 0.90, McDonald’s omega values from 0.60 to 0.90, and CR values from 0.60 to 0.91. Subjective Well-being demonstrated the strongest reliability (α = 0.90, ω = 0.90, CR = 0.91). Social Inclusion, Work Intensity, Professional Development, Health Risks, and Autonomy also showed acceptable-to-good reliability. In contrast, Workplace Harassment demonstrated comparatively modest reliability (α = 0.61, ω = 0.62, CR = 0.60), whereas Psychosomatic Symptoms showed borderline reliability (α = 0.59, ω = 0.60, CR = 0.61). These results should therefore be interpreted cautiously, particularly because these factors contain relatively few indicators.

In the held-out validation subsample, Cronbach’s alpha values ranged from 0.57 to 0.91, McDonald’s omega values from 0.61 to 0.91, and CR values from 0.68 to 0.92. Subjective Well-being again demonstrated the strongest reliability (α = 0.91, ω = 0.91, CR = 0.92). Social Inclusion, Work Intensity, Professional Development, Health Risks, and Autonomy also showed acceptable-to-good internal consistency and construct reliability. Workplace Harassment demonstrated comparatively low reliability (α = 0.57, ω = 0.61, CR = 0.68), while Psychosomatic Symptoms showed borderline reliability (α = 0.66, ω = 0.66, CR = 0.69). Overall, the reliability pattern observed in the held-out validation subsample was broadly comparable to that found in the calibration subsample.

In the calibration subsample, AVE values ranged from 0.41 to 0.76. AVE exceeded the recommended threshold of 0.50 for six of the eight factors. Social Inclusion showed an AVE of 0.41 despite good reliability (α = 0.88, ω = 0.88, CR = 0.87), whereas Work Intensity showed an AVE of 0.48. These findings indicate weaker convergence among the indicators of these two factors in the calibration subsample and therefore warrant cautious interpretation. The remaining factors demonstrated acceptable convergent validity, with AVE values ranging from 0.59 to 0.76.

In the held-out validation subsample, AVE values ranged from 0.40 to 0.77. AVE exceeded 0.50 for all factors except Social Inclusion (AVE = 0.40). Despite its lower AVE, Social Inclusion demonstrated good internal consistency and construct reliability (α = 0.87, ω = 0.87, CR = 0.87). The consistently lower AVE observed for Social Inclusion across both samples indicates comparatively weaker convergence among its indicators and may reflect, at least partly, the breadth and heterogeneity of this relational domain. The remaining factors demonstrated acceptable convergent validity, with AVE values ranging from 0.53 for Work Intensity to 0.77 for Psychosomatic Symptoms.

Taken together, the findings indicate a broadly comparable pattern of internal consistency, construct reliability, and convergent validity across the calibration and held-out validation subsamples. Nevertheless, psychometric performance was not uniformly strong across all factors. In particular, Workplace Harassment and Psychosomatic Symptoms demonstrated modest or borderline reliability, while Social Inclusion showed consistently lower AVE values in both samples. These domains should therefore be interpreted cautiously and may benefit from further item-level refinement and replication in future studies.

### 4.5. Discriminant Validity Across the Calibration and Held-Out Validation Subsamples

Discriminant validity was evaluated separately in the calibration subsample (N = 400) and the held-out validation subsample (N = 1231) using the heterotrait–monotrait ratio of correlations (HTMT). A conservative threshold of 0.85 was applied, with values below this threshold indicating adequate differentiation between the latent constructs.

In the calibration subsample, HTMT values ranged from 0.008 to 0.821. The highest value was observed between Subjective Well-being and Social Inclusion (HTMT = 0.821), followed by Subjective Well-being and Psychosomatic Symptoms (HTMT = 0.675), and Social Inclusion and Work Intensity (HTMT = 0.593). The lowest value was found between Psychosomatic Symptoms and Autonomy (HTMT = 0.008). Although the association between Subjective Well-being and Social Inclusion approached the conservative threshold, all HTMT values remained below 0.85, supporting adequate discriminant validity in thecalibration subsample (see Table 9).

In the held-out validation subsample, HTMT values ranged from 0.021 to 0.724. The highest value was again observed between Subjective Well-being and Social Inclusion (HTMT = 0.724), followed by Subjective Well-being and Psychosomatic Symptoms (HTMT = 0.636), and Work Intensity and Psychosomatic Symptoms (HTMT = 0.540). The lowest value was observed between Psychosomatic Symptoms and Autonomy (HTMT = 0.021). All HTMT values remained below 0.85, providing further support for the empirical distinctiveness of the eight MPWES factors in the held-out validation subsample.

Overall, the pattern of HTMT values was broadly comparable across the two subsamples. The results indicate that, despite several moderate associations between conceptually related factors, the eight MPWES dimensions were sufficiently distinct in both the calibration and held-out validation subsamples (see Table 10).

Because the calibration and held-out validation subsamples were randomly generated from the same dataset, these findings should be interpreted as evidence of internal split-sample measurement stability. They do not establish measurement invariance across independently recruited populations, organizations, employment sectors, cultures, or time points.

### 4.6. Measurement Invariance Across Samples

Measurement invariance between the calibration and held-out validation subsamples was examined using multi-group CFA with the WLSMV estimator. Configural, metric, scalar, and strict invariance models were tested sequentially to evaluate whether the refined eight-factor MPWES structure operated equivalently across the calibration and validation subsamples.

The configural model demonstrated acceptable fit (CFI = 0.919, TLI = 0.910, RMSEA = 0.065, SRMR = 0.070), supporting factorial equivalence across groups. Imposing equality constraints on factor loadings resulted in acceptable metric invariance (CFI = 0.931, TLI = 0.910, RMSEA = 0.059, SRMR = 0.071). Changes in approximate fit indices were minimal (ΔCFI = +0.013; ΔRMSEA = −0.006; ΔSRMR = +0.001), indicating that the relationships between observed indicators and latent constructs were comparable across the calibration and held-out validation subsamples.

Scalar invariance was subsequently tested by constraining item thresholds. The scalar model also demonstrated acceptable fit (CFI = 0.924, TLI = 0.902, RMSEA = 0.060, SRMR = 0.070), with negligible changes relative to the metric model (ΔCFI = −0.007; ΔRMSEA = +0.001; ΔSRMR < 0.001). These findings indicate that item thresholds were sufficiently comparable across the calibration and validation subsamples.

Finally, strict invariance was tested by additionally constraining residual variances. The strict invariance model maintained acceptable fit (CFI = 0.931, TLI = 0.921, RMSEA = 0.057, SRMR = 0.071; ΔCFI = +0.007; ΔRMSEA = −0.003; ΔSRMR = +0.001), indicating no meaningful deterioration in model fit after residual variances were constrained to equality across the two subsamples (see Table 11).

## 5. Discussion

The present study provides initial evidence regarding the structural validity, reliability, discriminant validity, and internal split-sample stability of the refined eight-factor MPWES model. The MPWES should be interpreted as a multidimensional psychosocial work experience measurement model rather than as a causal explanatory model or a measure of the psychosocial work environment alone. It integrates workplace demands and conditions, psychosocial resources, adverse exposures, health-related experiences, and subjective functioning as correlated but conceptually distinct first-order domains.

The findings provide internal psychometric evidence within the present dataset. However, external convergent validity, criterion validity, predictive validity, test–retest reliability, longitudinal invariance, and cross-cultural validation were not examined. Accordingly, the MPWES should be regarded as an instrument with promising initial structural evidence rather than as a fully validated tool for broad organizational, public health, or policy use.

### 5.1. Structural Validity and Theoretical Integration

The refined eight-factor model demonstrated acceptable global fit in both the calibration and held-out validation subsamples. The findings support interpreting the MPWES as a multidimensional psychosocial work experience profile composed of related but conceptually distinct occupational domains. However, the results do not support interpreting all eight domains as dimensions of one homogeneous psychosocial well-being construct [23,32].

In contrast to traditional psychosocial well-being instruments, which frequently examine job demands, job resources, and affective outcomes in analytically separate models [18,50], the present model evaluates these domains as related but distinct first-order factors within a correlated eight-factor measurement structure. This approach provides psychometric support for assessing these domains collectively as a multidimensional psychosocial work experience profile, without establishing a complete structural theory of occupational well-being [51].

Compared with established instruments, the MPWES should be interpreted as a complementary measure rather than a replacement. Instruments such as the Job Content Questionnaire and Effort–Reward Imbalance Questionnaire provide well-established assessments of specific job stress and work organization constructs [39,40], whereas the Job Satisfaction Survey, NIOSH WellBQ, and JD-R-based measures address job satisfaction, broader worker well-being, or demand–resource processes [15,17,41]. The MPWES differs in that it combines demand-related, resource-related, risk-related, symptom-related, and subjective functioning domains into a single correlated first-order measurement structure [34,35]. At the same time, the present findings do not indicate uniformly stronger psychometric performance than these established instruments. Some MPWES domains demonstrated good reliability and discriminant validity, whereas others showed modest reliability or lower AVE values and required further refinement. Therefore, the potential contribution of the MPWES lies in its integrated domain profile, while its reliability, validity evidence, scoring procedures, interpretability, and intended applications require further evaluation relative to established instruments [36,37,49].

The relatively low shared variance observed for the Social Inclusion dimension aligns with patterns reported in international research, where socially embedded and contextual constructs often reflect broader, more heterogeneous conceptual domains [52]. The lower AVE observed for Social Inclusion may partly reflect the conceptual breadth of this relational domain. However, it may also indicate item heterogeneity, weaker indicator convergence, or partial multidimensionality. Therefore, the result should not be attributed solely to construct breadth and warrants further item-level investigation [36,37]. However, while systemic integration enhances conceptual coherence, it may also limit the analytical disentanglement of dynamic causal pathways that are typically examined in longitudinal JD-R research.

### 5.2. Cross-Validation and Structural Stability

The refined eight-factor model demonstrated acceptable global fit in the held-out validation subsample without additional model modifications. The use of a held-out subsample is consistent with contemporary psychometric recommendations emphasizing internal split-sample validation as a method for reducing overfitting and evaluating structural reproducibility [36]. The acceptable global fit provides evidence of internal split-sample structural stability at the overall model level and is broadly consistent with previous multidimensional occupational measurement research reporting relatively stable demand- and resource-related factor structures [23,32].

However, the item-level structure was not fully reproduced. Specifically, the Work Intensity indicators V27 and V28 showed standardized loadings of 0.407 and 0.385, respectively, in the held-out validation subsample. Although job-resource dimensions such as Autonomy and Professional Development demonstrated comparatively stable indicator performance, broader or more heterogeneous occupational domains may show greater item-level variability [18,52].

The sensitivity analysis provided no evidence that removing V27 and V28 produced a clearly superior measurement model. In the calibration subsample, RMSEA decreased slightly, but SRMR increased to 0.086 and exceeded the prespecified threshold. In the held-out validation subsample, RMSEA remained unchanged, whereas CFI and TLI decreased and SRMR increased. Moreover, the residual covariance between V27 and V28 remained strong and statistically significant in both subsamples, with standardized estimates of 0.773 and 0.706. These findings indicate persistent local dependence and item-level instability within the Work Intensity factor. Therefore, acceptable global fit of the retained eight-factor model should not be interpreted as complete replication of the Work Intensity item structure. Further item development and evaluation in independently recruited samples are required.

### 5.3. Internal Consistency and Convergent Validity

The reliability findings indicate that most constructs demonstrate satisfactory internal consistency and structural coherence across samples. The overall pattern aligns with prior multidimensional psychosocial well-being instruments, in which composite reliability and omega coefficients generally meet recommended thresholds, even when constructs capture conceptually broad psychosocial domains [22,36]. The stability of reliability estimates across samples further strengthens confidence in the scale’s internal robustness.

The lower convergent validity observed for the Social Inclusion dimension reflects a pattern frequently reported in research on socially embedded constructs. In occupational and organizational psychology, inclusion, social climate, and relational support are often operationalized through heterogeneous indicators that capture multiple facets of interpersonal experience [52]. As a result, shared variance among indicators may be lower despite adequate overall reliability. Similar discrepancies between AVE and composite reliability have been documented in multidimensional workplace well-being scales, where conceptual breadth reduces average shared variance without undermining structural validity [36,37].

Reliability and convergent validity were not uniformly strong across all domains. Social Inclusion demonstrated good internal consistency and composite reliability but consistently low AVE values, indicating weaker convergence among its indicators. Work Intensity also showed an AVE below 0.50 in the calibration subsample, while Workplace Harassment and Psychosomatic Symptoms demonstrated modest or borderline reliability. These findings may partly reflect construct breadth, the small number of indicators, or the use of dichotomous items; however, they may also indicate item heterogeneity and insufficiently coherent measurement. These domains should therefore be interpreted cautiously and evaluated through further item refinement and replication.

These findings indicate that reliability and convergent validity evidence should be interpreted cautiously for some MPWES domains. In particular, the modest reliability estimates for Workplace Harassment and Psychosomatic Symptoms may reflect the small number of items and the use of risk-related or dichotomous indicators, but they also indicate the need for further replication. Similarly, the lower AVE values for Social Inclusion and Work Intensity suggest that these domains may require further conceptual and item-level refinement. Although the combination of Inclusion and Social Support into a broader Social Inclusion domain was theoretically and empirically justified, this refinement should be regarded as a step toward improved structural coherence rather than as definitive evidence that the domain is fully optimized.

### 5.4. Discriminant Validity and Construct Differentiation

The findings support adequate discriminant validity, indicating that the instrument’s constructs are empirically distinct despite their theoretical relatedness. However, evidence of discriminant validity should not be interpreted as evidence that all MPWES domains form a single homogeneous psychosocial well-being construct. Rather, low HTMT values indicate that the domains are empirically distinguishable. This supports interpreting the MPWES as a multidimensional profile comprising related yet distinct first-order domains. Accordingly, the instrument is intended to generate domain-specific profiles rather than a single overall score representing one unified latent construct. This is particularly important in multidimensional models of psychosocial well-being, where conceptually adjacent domains, such as job resources and subjective well-being, often show substantial intercorrelations [23]. The clear differentiation observed in the present study suggests that the domains represent structurally separable components of the multidimensional psychosocial work experience profile rather than redundant constructs.

The use of the HTMT ratio is consistent with contemporary structural equation modeling recommendations, which identify this approach as a more sensitive and reliable criterion for assessing discriminant validity than the traditional Fornell–Larcker procedure [49]. In JD-R–based research, partial construct overlap is frequently reported, particularly between demands, strain indicators, and affective outcomes [32]. Against this background, the present findings indicate successful operational differentiation of theoretically related but empirically distinct domains.

The high level of response completeness further supports the clarity and interpretability of the indicators. Psychometric literature emphasizes that discriminant validity reflects not only statistical separation but also conceptual precision in the definition of constructs [36].

### 5.5. Measurement Invariance and Structural Equivalence

Measurement invariance testing indicated no meaningful deterioration in model fit across configural, metric, scalar, and strict models for the calibration and held-out validation subsamples. Because these subsamples were generated by randomly splitting the same dataset, the findings should be interpreted as evidence of internal split-sample measurement stability rather than invariance across substantively distinct populations. They do not establish measurement equivalence across organizations, employment sectors, demographic groups, cultural contexts, or time points [53].

The confirmation of metric invariance indicates that the relationships between indicators and latent constructs are comparable across samples. Similar structural stability has been reported in Job Demands–Resources–based research, in which the resource and demand dimensions demonstrate consistency across occupational groups and contexts [23,32]. Establishing scalar invariance is particularly important, as it supports the interpretation that observed score differences reflect true differences in latent constructs rather than systematic measurement bias. In psychosocial well-being research, scalar invariance is not consistently achieved in heterogeneous samples, making this result methodologically noteworthy [53].

The additional support for strict invariance further indicates comparable measurement precision across samples. Such evidence is relatively uncommon among newly developed multidimensional scales and aligns with contemporary psychometric recommendations emphasizing greater invariance to ensure meaningful group comparisons [36]. This level of invariance suggests that the instrument may be suitable for future comparative research designs, although further invariance testing across demographic, occupational, and cultural groups is required before broader cross-sectoral applications can be recommended.

Taken together, the study primarily contributes by providing initial psychometric validation evidence for the refined eight-factor MPWES model in a large sample of employees. The study tested the model across calibration and held-out validation subsamples, evaluated reliability and discriminant validity, and examined measurement invariance across samples. These findings support the structural coherence and measurement stability of the MPWES in the present dataset. However, they should not be interpreted as evidence of policy-level utility or broad public health applicability. Additional validation studies using external criteria, predictive outcomes, longitudinal designs, and cross-cultural samples are needed before broader applications of the MPWES can be recommended.

### 5.6. Limitations of the Study

Despite the comprehensive psychometric evaluation, several methodological considerations warrant attention. First, the factorial structure was validated using cross-sectional data, which precludes direct conclusions regarding temporal stability, longitudinal invariance, or causal relationships among the examined dimensions. Although internal split-sample measurement stability was observed across the calibration and held-out validation subsamples, future longitudinal research is needed to examine stability over time and potential structural shifts in changing occupational environments.

Second, the present study relied exclusively on self-report measures. This approach is appropriate for assessing employees’ perceived psychosocial work environment and subjective well-being; however, it may increase the risk of response bias and common method bias. Because all variables were collected using the same survey format at a single time point, associations among constructs may partly reflect shared measurement method rather than substantive relationships. Future studies should therefore combine MPWES scores with external data sources, such as administrative records, sickness absence data, occupational health indicators, supervisor ratings, or established psychosocial work environment instruments.

Third, the present study did not include external validation criteria. Therefore, convergent validity with established instruments, criterion validity, predictive validity, and test–retest reliability were not examined. Future research should evaluate the MPWES in relation to established measures, including psychosocial work environment scales, well-being measures, burnout scales, work engagement instruments, and occupational health outcomes. Such studies are needed to determine whether MPWES scores predict relevant employee health, work-related, or organizational outcomes over time.

Fourth, participants were recruited using convenience sampling from only four organizations, with one organization representing each employment sector. Consequently, organization membership was fully confounded with sector membership, and organization-specific effects could not be separated from sector-specific effects. Because only four organizational clusters were available, within-organization dependence could not be reliably modeled using multilevel CFA or cluster-robust estimation. The findings therefore cannot establish generalizability across organizations or employment sectors, and the standard errors and factor estimates may have been affected by unmodeled clustering. Future studies should recruit multiple organizations within each sector and explicitly account for organizational clustering.

Fifth, measurement invariance across demographic and occupational groups was not examined in the present study. Although the refined MPWES model demonstrated measurement stability across the calibration and held-out validation subsamples, future studies should evaluate measurement invariance across gender, age groups, occupational sectors, occupations, and cultural contexts before the scale can be recommended for broader cross-sectoral comparisons or applied to occupational health assessment.

Sixth, some MPWES domains showed modest or borderline psychometric indicators. The lower AVEs observed for Social Inclusion and Work Intensity indicate that convergent validity for these domains should be interpreted with caution. In addition, V27 and V28 demonstrated standardized loadings below 0.50 in the held-out validation subsample, despite a residual covariance between these indicators having been introduced during calibration-stage refinement. This finding indicates that the Work Intensity item structure was not fully stable and requires further evaluation. Although the merging of Inclusion and Social Support into a broader Social Inclusion factor was theoretically and empirically justified, this domain may still require further conceptual and item-level refinement. In addition, Workplace Harassment and Psychosomatic Symptoms demonstrated modest or borderline reliability estimates, which may partly reflect the small number of items and the use of risk-related or dichotomous indicators. Future studies should examine whether additional or more specific items could improve the reliability, homogeneity, and convergent validity of these domains while preserving their conceptual relevance.

In addition, V27 and V28 demonstrated standardized loadings below 0.50 in the held-out validation subsample, despite a strong residual covariance between these indicators in both subsamples. A sensitivity analysis excluding V27 and V28 did not yield a consistently improved model and reduced the Work Intensity factor to only two indicators. These findings indicate item-level instability and possible local dependence within the Work Intensity factor, which requires further conceptual and item-level refinement.

Seventh, because the Financial Safety factor was excluded before the large-sample validation phase, the MPWES should not be interpreted as a comprehensive measure of all possible socioeconomic or financial determinants of employee well-being. Although financial security is relevant to broader well-being frameworks, the pilot findings did not support retaining it as a stable, reflective domain within the present MPWES structure. Future research may examine whether financial security can be assessed as a separate contextual or formative indicator alongside the MPWES domains.

Finally, the integrative modeling approach, in which job demands, resources, risks, health-related experiences, and subjective functioning are represented as related components of a multidimensional psychosocial work experience profile, prioritizes structural measurement over causal modeling. While this strengthens systemic conceptualization, it limits direct examination of directional pathways traditionally tested within JD-R frameworks. Future research may combine the present measurement architecture with structural path analyses to investigate causal mechanisms without fragmenting the multidimensional configuration.

### 5.7. Implications

#### 5.7.1. Theoretical Implications

The findings extend psychosocial well-being measurement by providing initial psychometric support for the MPWES as a multidimensional psychosocial work environment measurement model composed of related but distinct first-order domains. Rather than demonstrating a fully integrated theory of occupational well-being, the present study shows that job demand-related, resource-related, risk-related, symptom-related, and subjective functioning domains can be assessed within one correlated eight-factor measurement structure. By examining job demands, job resources, psychosocial risks, psychosomatic symptoms, and subjective functioning within a single empirically testable framework, the study contributes to multidimensional approaches that go beyond traditional predictor–outcome configurations [26].

The acceptable global fit of the refined eight-factor configuration across the calibration and held-out validation subsamples supports the structural coherence of the proposed measurement model in the present dataset. This integrative positioning is consistent with contemporary multidimensional perspectives on well-being that emphasize the interplay between contextual conditions and subjective functioning [21]. However, these findings should be interpreted as psychometric support for the proposed MPWES structure rather than as evidence of a complete theoretical model of psychosocial well-being at work.

#### 5.7.2. Methodological Implications

From a methodological perspective, the study illustrates the value of combining confirmatory factor analysis, split-sample validation, and measurement invariance testing in scale validation research. Evaluating the refined factor structure in an internal validation subsample reduces the risk of overfitting and strengthens confidence in structural reproducibility [36].

Establishing configural, metric, scalar, and strict invariance across calibration and held-out validation subsamples further indicates measurement stability within the internal split-sample design [54]. However, these findings do not establish measurement invariance across meaningful demographic or occupational groups, such as gender, age groups, sectors, occupations, or employment types. Moreover, the use of the WLSMV estimator and polychoric/tetrachoric correlation framework underscores the importance of appropriate estimation techniques when validating multidimensional instruments with ordinal and dichotomous indicators [36].

#### 5.7.3. Practical Implications for Organizations

From a practical perspective, the MPWES may be useful for describing domain-specific psychosocial work experience profiles in organizational and occupational health contexts. Rather than examining job demands, resources, risks, symptoms, and subjective functioning entirely separately, the instrument may help practitioners identify specific psychosocial domains that require further attention, including work intensity, autonomy, social inclusion, professional development, health risks, workplace harassment, psychosomatic symptoms, and subjective well-being.

The findings suggest that several MPWES domains demonstrated acceptable structural and reliability evidence in the present sample. However, the present study did not examine predictive validity, criterion validity, sensitivity to intervention, or longitudinal stability. Therefore, the MPWES should not yet be interpreted as an established tool for policy monitoring, intervention evaluation, workforce sustainability assessment, or organizational decision-making. These applications remain potential future uses that require additional validation in representative, longitudinal, occupationally diverse, and cross-cultural samples.

The demonstrated discriminant validity suggests that MPWES domains can be interpreted as distinct but related dimensions. This may support future domain-specific profiling and intervention planning, but such use should remain cautious until additional evidence confirms how MPWES scores relate to external occupational health outcomes, organizational indicators, and change over time.

#### 5.7.4. Policy and System-Level Implications

Given the model’s alignment with World Health Organization occupational health principles and OECD working environment indicators, the MPWES may have potential relevance for future occupational health monitoring and system-level research. However, the present study does not establish the instrument’s utility for national monitoring, policy evaluation, intervention assessment, or workforce sustainability evaluation. These applications remain potential future uses and require additional evidence, including external validation criteria, predictive validity, longitudinal stability, and validation in representative and culturally diverse samples.

At this stage, the findings primarily support the MPWES as a psychometric measurement model for assessing domain-specific components of a multidimensional psychosocial work experience profile. Future research may examine whether the instrument can contribute to organizational monitoring, sectoral comparison, or policy-related assessment, but such applications should be considered preliminary until further validation evidence is available.

## 6. Conclusions

This study examined the structural validity and internal split-sample measurement stability of the Multidimensional Psychosocial Work Experience Scale for Employed Persons (MPWES) in a large employee sample recruited from four organizations representing four employment sectors. The refined eight-factor model demonstrated acceptable global fit in both the calibration and held-out validation subsamples, adequate discriminant validity, and acceptable reliability for several domains. However, the item-level structure was not fully stable, particularly within the Work Intensity factor, and the sensitivity analysis excluding V27 and V28 did not provide a clearly superior solution. The MPWES is best interpreted as a multidimensional psychosocial work experience measurement model integrating workplace demands and conditions, psychosocial resources, adverse exposures, health-related experiences, and subjective functioning as correlated but conceptually distinct first-order domains.

The findings provide initial structural evidence rather than comprehensive validation. Item-level instability was observed within the Work Intensity factor, particularly for V27 and V28, and some domains demonstrated modest reliability or lower convergent validity. In addition, participants were recruited from only four organizations, with organization and employment sector fully confounded, and organization-level clustering was not modeled. The findings therefore do not establish generalizability or measurement invariance across organizations, employment sectors, cultures, or time points. Future studies should evaluate the MPWES in independently recruited, representative, longitudinal, organizationally diverse, and cross-cultural samples and examine its relationships with established measures and external occupational health outcomes before broader applied or policy-related use is recommended.

## Figures and Tables

**Table 2:** Demographic and occupational characteristics of the calibration and held-out validation subsample.

Characteristic	Total Sample (N = 1631)	Calibration Subsample (N = 400)	Held-Out Validation Subsample (N = 1231)	*p*-Value
Gender, n (%)				0.864
Women	1053 (64.6)	260 (65.0)	793 (64.4)	
Men	575 (35.3)	139 (34.8)	436 (35.4)	
Missing	3 (0.2)	1 (0.3)	2 (0.2)	
Age, years, mean (SD)	45.86 (11.90)	45.72 (11.84)	45.91 (11.92)	0.781
Length of service, years, mean (SD)	13.47 (11.83)	13.35 (11.78)	13.51 (11.85)	0.814
Employment sector, n (%)				1.000
Energy	489 (30.0)	120 (30.0)	369 (30.0)	
Pharmaceuticals	442 (27.1)	109 (27.3)	333 (27.1)	
Healthcare	341 (20.9)	84 (21.0)	257 (20.9)	
Administrative and support services	281 (17.2)	68 (17.0)	213 (17.3)	
Other or missing sector information	78 (4.8)	19 (4.8)	59 (4.8)	

Continuous variables are presented as mean and standard deviation; categorical variables are presented as frequency and percentage. Differences between samples were evaluated using an independent samples *t*-test for continuous variables and a chi-square test for categorical variables.

**Table 3:** Final MPWES domains, number of items, example item content, response formats, scoring direction, and theoretical source.

MPWES Domain	Number of Items	Example Item Content	Response Format	Scoring Direction	Theoretical Source
Subjective well-being	9	“I have felt cheerful and in good spirits.”	Six-point agreement/frequency scale	Higher scores indicate higher subjective well-being	WHO well-being; subjective well-being literature
Social Inclusion	15	“How often do you feel supported by your peers or colleagues?”	Six-point agreement/frequency scale	Higher scores indicate more favorable social inclusion and workplace support	WHO occupational health; OECD working environment; JD-R resources
Workplace harassment	4	“In the past 12 months, have you experienced workplace bullying or social exclusion?”	Dichotomous yes/no format	Reverse-coded so that higher scores indicate lower exposure to harassment	WHO occupational health; psychosocial risk literature
Work Intensity	4	“How often does your work involve working to tight deadlines?”	Six-point frequency scale	Reverse-coded so that higher scores indicate lower work intensity and more favorable conditions	JD-R demands; OECD working environment
Psychosomatic symptoms	2	“In the past 12 months, have you experienced mental health problems?”	Dichotomous yes/no format	Reverse-coded so that higher scores indicate fewer psychosomatic symptoms	WHO occupational health; occupational stress literature
Professional development	3	“In the past 12 months, have you received training that improved your job-related skills?”	Dichotomous yes/no format	Higher scores indicate greater access to professional development	JD-R resources; OECD working environment
Health risks	2	“How often are you exposed to noise at work?”	Six-point frequency scale	Reverse-coded so that higher scores indicate lower exposure to health risks	WHO occupational health; OECD working environment
Autonomy	2	“How often can you choose or change the methods of work?”	Six-point frequency scale	Higher scores indicate greater autonomy	JD-R resources; OECD working environment

Example items are presented in English translation. The Latvian version used in the present study is provided in Supplementary Material S2. Domain scores are calculated as the arithmetic mean of the corresponding items. Items 19, 20, 21, 22, 31, 32, 33, 34, 35, and 36 are reverse-coded before score computation to ensure directional consistency, so that higher domain scores consistently indicate a more favorable status within the corresponding occupational psychosocial domain.

**Table 4:** Statistical and theoretical justification for model refinement decisions.

Refinement Step	Statistical Reason	Theoretical Reason	Decision
Removal of V5	Standardized factor loading below 0.50	Weak empirical representation of the intended latent construct	Item removed
Removal of V11	Standardized factor loading below 0.50	Low item–factor association and limited contribution to construct measurement	Item removed
Merging Inclusion and Social Support	Conceptual overlap and empirical proximity between factors	Both domains reflect relational and social workplace resources	Combined into Social Inclusion
Residual covariance V27–V28	Shared residual variance between semantically related items	Items reflected closely related content within the same conceptual domain	Residual covariance added
Residual covariance V18–V3	Shared residual variance between semantically overlapping indicators	Items captured related aspects of the same psychosocial experience	Residual covariance added
Residual covariance V14–V15	Shared residual variance between closely related items	Items represented similar content within the same relational workplace domain	Residual covariance added

**Table 5:** Latent factor correlations in the calibration subsample (N = 400).

Factor	1	2	3	4	5	6	7	8
1. Subjective Well-being	1.000							
2. Social Inclusion	0.802	1.000						
3. Workplace Harassment	0.277	0.395	1.000					
4. Work Intensity	−0.649	−0.678	−0.448	1.000				
5. Psychosomatic Symptoms	0.635	0.424	0.541	−0.618	1.000			
6. Professional Development	−0.311	−0.512	−0.115	0.397	−0.095	1.000		
7. Health Risks	−0.240	−0.391	−0.409	0.553	−0.178	0.358	1.000	
8. Autonomy	0.357	0.500	0.148	−0.305	0.031	−0.263	−0.268	1.000

Values represent standardized latent factor correlations estimated in the refined eight-factor CFA model. Correlations are presented below the diagonal.

**Table 6:** Latent factor correlations in the held-out validation subsample (N = 1231).

Factor	1	2	3	4	5	6	7	8
1. Subjective Well-being	1.000							
2. Social Inclusion	0.684	1.000						
3. Workplace Harassment	0.346	0.584	1.000					
4. Work Intensity	−0.540	−0.469	−0.644	1.000				
5. Psychosomatic Symptoms	0.651	0.429	0.577	−0.610	1.000			
6. Professional Development	−0.135	−0.209	0.152	−0.010	0.075	1.000		
7. Health Risks	−0.097	−0.199	−0.388	0.432	−0.091	−0.083	1.000	
8. Autonomy	0.203	0.351	0.340	−0.196	0.028	−0.109	−0.347	1.000

Values represent standardized latent factor correlations estimated in the refined eight-factor CFA model. Correlations are presented below the diagonal.

**Table 7:** Residual covariance estimates in the calibration and held-out validation subsamples.

Item Pair	Calibration Estimate	SE	*p*-Value	Standardized	Validation Estimate	SE	*p*-Value	Standardized
V27–V28	0.501	0.041	<0.001	0.773	0.597	0.020	<0.001	0.706
V18–V3	0.370	0.034	<0.001	0.612	0.342	0.021	<0.001	0.610
V14–V15	0.315	0.029	<0.001	0.501	0.248	0.016	<0.001	0.419

Estimates represent unstandardized residual covariances. Standardized values are reported in the corresponding columns. All residual covariance estimates were statistically significant at *p* < 0.001.

**Table 8:** Reliability and convergent validity indicators of the MPWES factors in the calibration and held-out validation subsample.

Factor	Calibration Subsample (N = 400)				Held-Out Validation Subsample (N = 1231)			
	Cronbach’s α	McDonald’s ω	CR	AVE	Cronbach’s α	McDonald’s ω	CR	AVE
Subjective Well-being	0.90	0.90	0.91	0.59	0.91	0.91	0.92	0.62
Social Inclusion	0.88	0.88	0.87	0.41	0.87	0.87	0.87	0.40
Workplace Harassment	0.61	0.62	0.60	0.64	0.57	0.61	0.68	0.66
Work Intensity	0.78	0.78	0.70	0.48	0.84	0.85	0.73	0.53
Psychosomatic Symptoms	0.59	0.60	0.61	0.67	0.66	0.66	0.69	0.77
Professional Development	0.78	0.79	0.79	0.76	0.77	0.79	0.80	0.74
Health Risks	0.79	0.80	0.81	0.76	0.83	0.83	0.77	0.70
Autonomy	0.79	0.79	0.79	0.69	0.81	0.82	0.81	0.73

α = Cronbach’s alpha; ω = McDonald’s omega; CR = composite reliability; AVE = average variance extracted. Values of α, ω, and CR ≥ 0.70 were interpreted as indicating acceptable reliability, whereas AVE ≥ 0.50 was considered evidence of acceptable convergent validity. Values below conventional thresholds should be interpreted cautiously.

**Table 9:** Heterotrait–monotrait ratios of correlations for the MPWES factors in the calibration subsample (N = 400).

Factor	1	2	3	4	5	6	7	8
Subjective well-being	1.000							
Social inclusion	0.821	1.000						
Workplace harassment	0.270	0.287	1.000					
Work intensity	0.572	0.593	0.342	1.000				
Psychosomatic symptoms	0.675	0.389	0.552	0.475	1.000			
Professional development	0.200	0.443	0.075	0.336	0.151	1.000		
Health risks	0.324	0.410	0.372	0.478	0.314	0.203	1.000	
Autonomy	0.277	0.436	0.117	0.226	0.008	0.268	0.214	1.000

HTMT = heterotrait–monotrait ratio of correlations. Values below 0.85 were considered evidence of adequate discriminant validity. The lower triangular matrices are presented to avoid duplication.

**Table 10:** Heterotrait–monotrait ratios of correlations for the MPWES factors in the held-out validation subsample (N = 1231).

Factor	1	2	3	4	5	6	7	8
Subjective well-being	1.000							
Social inclusion	0.724	1.000						
Workplace harassment	0.294	0.450	1.000					
Work intensity	0.403	0.295	0.496	1.000				
Psychosomatic symptoms	0.636	0.405	0.443	0.540	1.000			
Professional development	0.097	0.153	0.060	0.042	0.047	1.000		
Health risks	0.069	0.143	0.320	0.328	0.024	0.044	1.000	
Autonomy	0.161	0.296	0.267	0.197	0.021	0.105	0.415	1.000

HTMT = heterotrait–monotrait ratio of correlations. Values below 0.85 were considered evidence of adequate discriminant validity. The lower triangular matrices are presented to avoid duplication.

**Table 11:** Measurement invariance testing across calibration and held-out validation subsamples.

Model	CFI	TLI	RMSEA	SRMR	ΔCFI	ΔRMSEA	ΔSRMR
Configural invariance	0.919	0.910	0.065	0.070	—	—	—
Metric invariance	0.931	0.910	0.059	0.071	+0.013	−0.006	+0.001
Scalar invariance	0.924	0.902	0.060	0.070	−0.007	+0.001	<0.001
Strict invariance	0.931	0.921	0.057	0.071	+0.007	−0.003	+0.001

Note. Measurement invariance was evaluated using changes in approximate fit indices, including CFI, RMSEA, and SRMR. All changes remained within commonly accepted limits for nested invariance models, indicating internal split-sample stability at the configural, metric, scalar, and strict levels across the calibration and held-out validation subsamples. These results should not be interpreted as evidence of invariance across occupational sectors or externally recruited samples.

## Data Availability

Due to ethical and confidentiality considerations, the dataset analysed in this study is not publicly available. Anonymised data may be made available by the corresponding author upon reasonable request and in accordance with institutional regulations.

## Supplementary Materials

The following supporting information can be downloaded at https://www.mdpi.com/article/10.3390/ijerph23081080/s1, Material S1: English version of the Multidimensional Psychosocial Work Experience Scale for Employed Persons (MPWES). Material S2: Latvian version of the Multidimensional Psychosocial Work Experience Scale for Employed Persons (MPWES). Material S3: Item-level CFA results, modification indices, and detailed latent factor correlations for the calibration and held-out validation subsample.

## Abbreviations

The following abbreviations are used in this manuscript:

MPWES	Multidimensional Psychosocial Work Experience Scale for Employed Persons (MPWES)
AVE	Average Variance Extracted
CFA	Confirmatory Factor Analysis
CFI	Comparative Fit Index
CR	Composite Reliability
HTMT	Heterotrait–Monotrait Ratio of Correlations
JD-R	Job Demands–Resources
RMSEA	Root Mean Square Error of Approximation
SRMR	Standardized Root Mean Square Residual
TLI	Tucker–Lewis Index
WHO	World Health Organization
