# Supplementary material for: Psychosocial Well-Being at Work: Large-Sample Structural Validation of the Multidimensional Psychosocial Work Experience Scale for Employed Persons (MPWES)
Source: Int J Environ Res Public Health. 2026 Aug 19;23(8):1080. doi: 10.3390/ijerph23081080 (PMC13513784; doi:10.3390/ijerph23081080)
Supplement: Supplementary file 1 [file ijerph-23-01080-s001.zip › Supplementary Materials S1_English Version of the MPWES.pdf]

Multidimensional Psychosocial Work Environment Scale for Employed Persons (MPWES)

This document includes the following information:

1. Instructions for completing the questionnaire.

2. Questionnaire items.

3. Evaluation of the survey results.

4. Item grouping according to the factors.

PLEASE READ THE QUESTIONNAIRE INSTRUCTIONS FIRST

The questionnaire contains statements about how people tend to feel in their day-to-day life at the workplace. Please tick one answer per line. Read each statement/question and rate how much you agree or disagree with each statement.

Please indicate the extent to which you agree or disagree with the statements about your personal experience at work.

Tick one answer per line

1 Strongly disagree	2 Disagree	3 Rather disagree	4 Rather agree	5 Agree	6 Strongly agree

1	Overall, I am satisfied with my professional life	1	2	3	4	5	6
2	I trust my colleagues in this organization	1	2	3	4	5	6
3	I trust my direct manager	1	2	3	4	5	6
4	I am satisfied with my social relationships at work	1	2	3	4	5	6
5	I am satisfied with my physical health	1	2	3	4	5	6
6	I am satisfied with the amount of time I have to do the things I enjoy	1	2	3	4	5	6
7	I feel that my work contributes positively to society	1	2	3	4	5	6
8	My job offers good prospects for career advancement	1	2	3	4	5	6
9	I am satisfied with the organization I work for	1	2	3	4	5	6

Please indicate how often you experience the following in the workplace.

Tick one answer per line

1<br>At no time	2<br>Sometimes	3<br>Less than half the time	4<br>More than half the time	5<br>Most time	6<br>All the time

10	How often does your job give you a sense of work well done?	**1**	**2**	**3**	**4**	**5**	**6**
11	How often do you feel your life is worthwhile?	1	2	3	4	5	6
12	How often do you feel you are involved in improving work processes?	1	2	3	4	5	6
13	How often do you feel you can influence decisions that are important to your work?	1	2	3	4	5	6
14	How often do you trust your executive management?	1	2	3	4	5	6
15	How often do you feel supported by your peers or colleagues?	1	2	3	4	5	6
16	How often do you feel supported by your manager?	1	2	3	4	5	6
17	How often does your main paid job involve learning new things?	1	2	3	4	5	6
18	How often do you feel your life is in balance?	1	2	3	4	5	6
19	How often are you exposed to chemicals at work?	1	2	3	4	5	6
20	How often are you exposed to noise at work?	1	2	3	4	5	6
21	How often do you feel emotionally drained by your work?	1	2	3	4	5	6
22	How often do you feel physically exhausted at the end of your workday?	1	2	3	4	5	6
23	How often can you choose or change the methods of work?	1	2	3	4	5	6
24	How often can you choose or change the order of your own tasks?	1	2	3	4	5	6
25	How often does your work involve working to tight deadlines?	1	2	3	4	5	6
26	How often does your work involve working at high speed?	1	2	3	4	5	6
27	How often do you receive the recognition you deserve for your work?	1	2	3	4	5	6

Please indicate whether you have had any of the following experiences at work in the last year.

Tick one answer per line

1<br>Yes	2<br>No

28	In the past 12 months, have you received training paid for or provided by your employer?	1	2
29	In the past 12 months, have you received training aimed at improving your future work prospects?	1	**2**
30	In the past 12 months, have you received training that improved your job-related skills?	1	**2**
31	In the past 12 months, have you faced unwanted sexual attention at your work?	1	**2**
32	In the past 12 months, have you faced verbal abuse or threats at your work?	1	**2**
33	In the past 12 months, have you experienced workplace bullying or social exclusion?	1	**2**
34	In the past 12 months, have you faced discrimination at your work?	1	**2**
35	In the past 12 months, have you experienced mental health problems?	1	**2**
36	In the past 12 months, have you experienced physical health problems?	1	**2**

Please indicate how often you experience the following in the workplace.

Tick one answer per line

1<br>At no time	2<br>Sometimes	3<br>Less than half the time	4<br>More than half the time	5<br>Most time	6<br>All the time

In the last two weeks...							
37	I have felt cheerful and in good spirits	1	2	3	4	5	6
38	I have felt calm and relaxed	1	2	3	4	5	6
39	I have felt active and vigorous	1	2	3	4	5	6
40	I woke up feeling fresh and rested	1	2	3	4	5	6
41	My daily life has been filled with things that interest me	1	2	3	4	5	6

Thank you very much!

- ✓ *Survey guidelines:*
- ✓ **Items to be reversed:**

**Items**	**19, 20, 21, 22**	**28, 29, 30**	**31, 32, 33, 34, 35, 36**
**Recoding**	1 = 6	1 = 2	No recoding
	2 = 5	2 = 1	1 = Yes
	3 = 4		2 = No
	4 = 3		

	5 = 2		
	6 = 1		

- ✓ The arithmetic average *(Mean)* of the corresponding items is used to determine the value of each well-being factor *(Mean)*.

Factors	*Related items*	*Number of items*
**1. Subjective well-being**	5,6, 11,18, 37, 38, 39, 40, 41	9
**2. Social Inclusion**	1, 2,3,4,7,8 9, 10, 12, 13, 14, 15,16, 17, 27	15
**3. Workplace harassment**	31,32,33,34	4
**4. Work intensity**	21,22,25,26	4
**5. Psychosomatic symptoms**	35,36	2
**6. Professional development**	28,29,30	3
**7. Health risks**	19,20	2
**8. Autonomy**	23,24	2
