# Supplementary material for: Psychosocial Well-Being at Work: Large-Sample Structural Validation of the Multidimensional Psychosocial Work Experience Scale for Employed Persons (MPWES)
Source: Int J Environ Res Public Health. 2026 Aug 19;23(8):1080. doi: 10.3390/ijerph23081080 (PMC13513784; doi:10.3390/ijerph23081080)
Supplement: Supplementary file 1 [file ijerph-23-01080-s001.zip › Supplementary Materials S2_Latvian Version of the MPWES.pdf]

Psihosociālās labbūtības daudzdimensionālā skala nodarbinātajām personām / Multidimensional Psychosocial Work Environment Scale for Employed Persons (MPWES)

Šis dokuments iekļauj sekojošo informāciju:

1. Aptaujas aizpildīšanas instrukcija.

2. Aptaujas vienumi.

3. Aptaujas rezultātu novērtēšana.

4. Vienumu grupēšana atbilstoši faktoriem.

LŪDZU, VISPIRMS IZLASIET APTAUJAS INSTRUKCIJU

Aptaujā ir iekļauti apgalvojumi par to, kā cilvēki mēdz justies ikdienā darba vietā. Lūdzu, atzīmējiet vienu atbildi katrā rindā. Izlasiet katru apgalvojumu/jautājumu un novērtējiet, cik lielā mērā piekrītat vai nepiekrītat katram apgalvojumam.

Lūdzu, atzīmējiet, cik lielā mērā piekrītat vai nepiekrītat apgalvojumiem par Jūsu personīgo pieredzi darbā

Atzīmējiet vienu atbildi katrā rindā

1 Nepiekrītu nemaz	2 Nepiekrītu	3 Drīzāk nepiekrītu	4 Drīzāk piekrītu	5 Piekrītu	6 Pilnīgi piekrītu

1	Ar savu profesionālo dzīvi kopumā esmu apmierināts/a	1	2	3	4	5	6
2	Es uzticos citiem mūsu organizācijas darbiniekiem	1	2	3	4	5	6
3	Es uzticos savam tiešajam vadītājam	1	2	3	4	5	6
4	Esmu apmierināts/a ar savstarpējo saskarsmi darba vietā	1	2	3	4	5	6
5	Esmu apmierināts/a esat ar savu fizisko veselību	1	2	3	4	5	6
6	Esmu apmierināts/a ar to, cik daudz laika varu veltīt man interesantām, patīkamām lietām, nodarbēm ārpus darba	1	2	3	4	5	6
7	Mans darbs sniedz pozitīvu ieguldījumu sabiedrībā	1	2	3	4	5	6
8	Mans darbs piedāvā labas karjeras izaugsmes iespējas	1	2	3	4	5	6
9	Ar darbu šajā organizācijā kopumā esmu apmierināts/-a	1	2	3	4	5	6

Lūdzu, atzīmējiet, cik bieži Jūs saskaraties ar attiecīgo pieredzi darbā

Atzīmējiet vienu atbildi katrā rindā

1 Nevienā brīdī	2 Dažkārt	3 Mazāk nekā puse laika	4 Vairāk nekā puse laika	5 Lielāko daļu laiku	6 Visu laiku

10	Cik bieži šajā darbā Jums ir labi padarīta darba sajūta?	**1**	**2**	**3**	**4**	**5**	**6**
11	Cik bieži Jums ir sajūta, ka dzīvojat pilnvērtīgu dzīvi?	1	2	3	4	5	6
12	Cik bieži Jūs jūtaties iesaistīts/a darba procesu uzlabošanā savā organizācijā/komandā?	1	2	3	4	5	6
13	Cik bieži Jums šķiet, ka varat ietekmēt lēmumus, kas ir svarīgi Jūsu darbā?	1	2	3	4	5	6
14	Cik lielā mērā Jūs uzticaties savas organizācijas augstākajai vadībai?	1	2	3	4	5	6
15	Cik bieži Jūs jūtaties savu kolēģu atbalstīts/a?	1	2	3	4	5	6
16	Cik bieži Jūs jūtaties sava vadītāja atbalstīts/a?	1	2	3	4	5	6
17	Cik bieži Jūs savā darbā apgūstat ko jaunu?	1	2	3	4	5	6
18	Cik bieži jūtat, ka Jūsu dzīve ir līdzsvarā (darbs un personīgais laiks)?	1	2	3	4	5	6
19	Cik bieži Jūs esat pakļauts/a ķīmisko vielu iedarbībai darbā?	1	2	3	4	5	6
20	Cik bieži Jūs esat pakļauts/a trokšņa iedarbībai darbā?	1	2	3	4	5	6
21	Cik bieži Jūs jūtaties emocionāli iztukšots/a sava darba dēļ?	1	2	3	4	5	6
22	Cik bieži Jūs darba dienas beigās jūtaties fiziski noguris/usi?	1	2	3	4	5	6
23	Cik bieži Jūs varat pats/i izvēlēties sava darba metodes vai tās mainīt pēc saviem ieskatiem?	1	2	3	4	5	6
24	Cik bieži Jūs varat pats/i izvēlēties savu darba uzdevumu secību vai mainīt to pēc saviem ieskatiem?	1	2	3	4	5	6
25	Cik bieži Jūsu darbs pieprasa uzdevumu izpildi ļoti saspringtos, īsos termiņos?	1	2	3	4	5	6
26	Cik bieži Jums jāstrādā straujā tempā, lielā ātrumā?	1	2	3	4	5	6
27	Cik bieži Jūs saņemat atzinību, uzslavu par labi padarītu darbu?	1	2	3	4	5	6

Lūdzu, atzīmējiet, vai pagājušajā gadā darbā saskārāties vai nesaskārāties ar attiecīgo pieredzi

Atzīmējiet vienu atbildi katrā rindā

1<br>Jā	2<br>Nē

28	Pagājušajā gadā esmu piedalījies/usies apmācībās, kuras apmaksājis vai nodrošinājis mans darba devējs	1	2
29	Pēdējā gada laikā esmu saņēmis/usi apmācības, kas uzlabo manas turpmākās darba izredzes	1	2
30	Pēdējā gadā laikā esmu saņēmis/usi apmācības, kas uzlaboja manas prasmes	1	2
31	Pēdējā gada laikā esmu saskāries/usies ar iebiedēšanu, uzmākšanos vai vardarbību savā darbā	1	2
32	Pēdējā gada laikā esmu saskāries/usies ar verbālu (vārdisku) vardarbību vai draudiem darbā	1	2
33	Pēdējā gada laikā esmu saskāries/usies ar emocionālu izstumšanu (piemēram, mobingu) darbā	1	2
34	Pēdējā gada laikā esmu saskāries/usies ar diskrimināciju darbā	1	2
35	Pēdējā gada laikā esmu sev novērojis/usi psihiskās veselības traucējumus	1	2
36	Pēdējā gada laikā esmu sev novērojis/usi fiziskās veselības traucējumus	1	2

Lūdzu, atzīmējiet, cik bieži Jūs saskaraties ar attiecīgo pieredzi darbā

Atzīmējiet vienu atbildi katrā rindā

1<br>Nevienā brīdī	2<br>Dažkārt	3<br>Mazāk nekā puse laika	4<br>Vairāk nekā puse laika	5<br>Lielāko daļu laiku	6<br>Visu laiku

Pēdējo divu nedēļu laikā…

37	biju priecīgs/a un labā noskaņojumā	1	2	3	4	5	6
38	biju juties/usies mierīgi un atbrīvoti	1	2	3	4	5	6
39	biju juties/usies enerģiski un aktīvi	1	2	3	4	5	6
40	pēc pamošanās jutos mundrs/s un atpūties/usies	1	2	3	4	5	6
41	mana ikdiena bija piepildīta ar lietām, kas mani interesē	1	2	3	4	5	6

Liels paldies!

Reversējamie vienumi:

**Vienums**	**19, 20, 21, 22**	**28, 29, 30**	**31, 32, 33, 34, 35, 36**
**Kodēšana**	**1 = 6**	**1 = 2**	**Neveic kodēšnu**
	**2 = 5**	**2 = 1**	**1 = Jā**
	**3 = 4**		**2 = Nē**
	**4 = 3**		
	**5 = 2**		
	**6 = 1**		

Katra labbūtības faktora vērtības noteikšanā tiek izmantots atbilstošo vienumu vidējais aritmētiskais *(Mean).*

Factors	***Vienumi***	Vienumu skaits
**1.Subjektīvā labbūtība**	5,6, 11,18, 37, 38, 39, 40, 41	9
**2.Sociālā iekļaušana**	1, 2,3,4,7,8 9, 10, 12, 13, 14, 15,16, 17, 27	15
**3.Psihosociālais apdraudējums**	31,32,33,34	4
**4. Darba intensitātē**	21,22,25,26	4
**5. Psihosomatika**	35,36	2
**6. Profesionālā izaugsme**	28,29,30	3
**7. Veselības riski**	19,20	2
**8. Autonomija**	23,24	2
