# Supplementary material for: Psychosocial Well-Being at Work: Large-Sample Structural Validation of the Multidimensional Psychosocial Work Experience Scale for Employed Persons (MPWES)
Source: Int J Environ Res Public Health. 2026 Aug 19;23(8):1080. doi: 10.3390/ijerph23081080 (PMC13513784; doi:10.3390/ijerph23081080)
Supplement: Supplementary file 1 [file ijerph-23-01080-s001.zip › Supplementary Materials S3.pdf]

Supplementary Table S1. Item-level factor loadings for the refined eight-factor MPWES model in the calibration sample (N = 400).

Factor	Item	Unstandardized estimate (B)	SE	p	Standardized loading (λ)
Subjective well-being	V6	1.000	—	—	0.624
Subjective well-being	V7	1.163	0.058	< 0.001	0.726
Subjective well-being	V13	1.243	0.065	< 0.001	0.776
Subjective well-being	V20	1.334	0.070	< 0.001	0.833
Subjective well-being	V39	1.351	0.068	< 0.001	0.843
Subjective well-being	V40	1.314	0.067	< 0.001	0.821
Subjective well-being	V41	1.370	0.067	< 0.001	0.855
Subjective well-being	V42	1.231	0.066	< 0.001	0.768
Subjective well-being	V43	1.337	0.068	< 0.001	0.834
Social inclusion	V1	1.000	—	—	0.694
Social inclusion	V2	0.877	0.059	< 0.001	0.609
Social inclusion	V4	0.884	0.056	< 0.001	0.613
Social inclusion	V8	0.870	0.060	< 0.001	0.604
Social inclusion	V10	1.044	0.055	< 0.001	0.724
Social inclusion	V17	0.896	0.055	< 0.001	0.622
Social inclusion	V9	0.836	0.062	< 0.001	0.580
Social inclusion	V12	0.925	0.053	< 0.001	0.642
Social inclusion	V14	0.832	0.064	< 0.001	0.577
Social inclusion	V15	0.894	0.061	< 0.001	0.620
Social inclusion	V16	0.973	0.063	< 0.001	0.675
Social inclusion	V18	0.996	0.059	< 0.001	0.691
Social inclusion	V29	0.792	0.066	< 0.001	0.549
Social inclusion	V3	0.869	0.454	< 0.001	0.540
Social inclusion	V19	0.912	0.021	< 0.001	0.614
Workplace harassment	V34	1.000	—	—	0.676
Workplace harassment	V35	1.442	0.305	< 0.001	0.975
Workplace harassment	V36	1.107	0.228	< 0.001	0.749

Factor	Item	Unstandardized estimate (B)	SE	p	Standardized loading (λ)
Workplace harassment	V33	1.001	0.134	< 0.001	0.698
Work intensity	V23	1.000	—	—	0.920
Work intensity	V24	0.822	0.040	< 0.001	0.757
Work intensity	V27	0.658	0.046	< 0.001	0.606
Work intensity	V28	0.639	0.047	< 0.001	0.588
Psychosomatic symptoms	V37	1.000	—	—	0.889
Psychosomatic symptoms	V38	0.971	0.084	< 0.001	0.863
Professional development	V30	1.000	—	—	0.579
Professional development	V31	1.574	0.203	< 0.001	0.912
Professional development	V32	1.783	0.230	< 0.001	0.921
Health risks	V21	1.000	—	—	0.997
Health risks	V22	1.103	0.124	< 0.001	0.879
Autonomy	V25	1.000	—	—	0.865
Autonomy	V26	0.972	0.081	< 0.001	0.841

Note. The first item of each factor was used as the reference indicator, and its unstandardized loading was fixed to 1.000; therefore, no standard error or p-value was estimated for these items. SE = standard error. All freely estimated factor loadings were statistically significant at $p < 0.001$.

Supplementary Table S2. Modification indices for the residual covariances retained in the refined MPWES model:based on the calibration sample structure (N = 400).

Item 1	Operator	Item 2	MI	EPC	sepc.lv	sepc.all	sepc.nox
V27	~~	V28	31,84221	0,18465404	0,18492054	0,4120983	0,4120321
V18	~~	V3	22,61586	0,15647432	0,15686104	0,3467636	0,3468766
V14	~~	V15	17,90343	0,13997432	0,13975213	0,3017630	0,3019303

Note. MI = modification index; EPC = expected parameter change; sepc.lv = standardized expected parameter change for latent variables; sepc.all = completely standardized expected parameter change; sepc.nox = standardized expected parameter change excluding exogenous variables.

Supplementary Table S3. Principal modification indices for the initial nine-factor MPWES model in the calibration sample (N = 400).

Item 1	op	Item 2	MI	EPC	sepc.lv	sepc.all	sepc.nox
Subjective_WB	=~	V1	120,15719	1,0418625	0,6504009	0,6504009	0,6504009
Subjective_WB	=~	V12	90,337458	0,8959383	0,5593052	0,5593052	0,5593052
V41	~~	V42	84,232906	0,2105634	0,2105634	0,634672	0,634672
V7	~~	V20	75,759737	0,2288413	0,2288413	0,6012705	0,6012705
V6	~~	V38	72,101383	0,3639157	0,3639157	0,9230254	0,9230254
V13	~~	V1	63,016695	0,2665577	0,2665577	0,5866635	0,5866635
Health_Risk	=~	V10	61,792499	-0,4047285	-0,322538	-0,322538	-0,322538
Symptoms	=~	V6	58,636447	0,4510956	0,4011485	0,4011485	0,4011485
Prof_Dev	=~	V12	45,098946	0,6458555	0,3740068	0,3740068	0,3740068
Subjective_WB	=~	V30	45,055031	0,3553383	0,2218262	0,2218262	0,2218262
Intensity	=~	V41	44,869391	0,33386	0,3072237	0,3072237	0,3072237
Subjective_WB	=~	V10	44,297569	-0,6370088	-0,3976639	-0,3976639	-0,3976639
Intensity	=~	V10	43,964557	-0,3772252	-0,3471292	-0,3471292	-0,3471292
Social_Inclusion	=~	V30	42,910544	0,3706866	0,2571955	0,2571955	0,2571955
Symptoms	=~	V30	39,57829	0,2926947	0,2602864	0,2602864	0,2602864
Social_Inclusion	=~	V42	38,652146	-0,5178108	-0,3592755	-0,3592755	-0,3592755
Prof_Dev	=~	V1	38,537854	0,6083139	0,3522669	0,3522669	0,3522669
V2	~~	V4	38,423179	0,2397752	0,2397752	0,3825821	0,3825821
Prof_Dev	=~	V10	36,679439	-0,5093368	-0,2949505	-0,2949505	-0,2949505
Health_Risk	=~	V41	33,950711	0,2786521	0,2220647	0,2220647	0,2220647
Social_Inclusion	=~	V41	33,506074	-0,4597019	-0,3189575	-0,3189575	-0,3189575
Intensity	=~	V30	32,797957	-0,2609255	-0,2401082	-0,2401082	-0,2401082
V31	~~	V32	31,168859	-0,5649724	-0,5649724	-5,3495005	-5,3495005
Symptoms	=~	V1	31,044642	0,2880381	0,2561454	0,2561454	0,2561454
Social_Inclusion	=~	V31	30,783862	-0,4871421	-0,3379965	-0,3379965	-0,3379965
Harassment	=~	V10	30,700715	0,4402301	0,2977676	0,2977676	0,2977676
Autonomy	=~	V42	29,744451	-0,285427	-0,2468803	-0,2468803	-0,2468803
Harassment	=~	V6	29,374618	0,3724903	0,2519491	0,2519491	0,2519491
Symptoms	=~	V12	28,966108	0,2749784	0,2445317	0,2445317	0,2445317
Health_Risk	=~	V12	28,598988	0,3028813	0,2413736	0,2413736	0,2413736

Intensity	=~	V14	28,468537	0,3448305	0,317319	0,317319	0,317319
Harassment	=~	V41	27,683509	-0,3935799	-0,2662138	-0,2662138	-0,2662138
Symptoms	=~	V14	26,755677	-0,2831485	-0,2517972	-0,2517972	-0,2517972
Subjective_WB	=~	V31	25,551434	-0,37614	-0,234812	-0,234812	-0,234812
Prof_Dev	=~	V41	24,468539	0,3620328	0,2096486	0,2096486	0,2096486
Subjective_WB	=~	V29	24,424389	-0,5397793	-0,3369667	-0,3369667	-0,3369667
V20	~~	V41	24,24641	-0,1855678	-0,1855678	-0,6464888	-0,6464888
V17	~~	V18	24,055942	0,1713227	0,1713227	0,302723	0,302723
Autonomy	=~	V15	22,729202	0,2564248	0,2217948	0,2217948	0,2217948
V10	~~	V23	22,301804	-0,1781036	-0,1781036	-0,6601419	-0,6601419
V41	~~	V28	22,148896	0,2309118	0,2309118	0,5504915	0,5504915
Prof_Dev	=~	V42	21,90166	0,3551682	0,2056734	0,2056734	0,2056734
Social_Inclusion	=~	V43	21,735193	0,3678104	0,2551999	0,2551999	0,2551999
V6	~~	V1	21,600648	0,1827799	0,1827799	0,3248982	0,3248982
V30	~~	V32	21,577737	0,3166875	0,3166875	1,5121128	1,5121128
Intensity	=~	V20	21,535587	-0,2258191	-0,2078027	-0,2078027	-0,2078027
Symptoms	=~	V15	21,451372	-0,2480536	-0,2205881	-0,2205881	-0,2205881
Prof_Dev	=~	V17	21,008206	0,4539565	0,2628805	0,2628805	0,2628805
Health_Risk	=~	V20	20,888309	-0,2160819	-0,172201	-0,172201	-0,172201
Harassment	=~	V30	20,26751	0,3854225	0,2606963	0,2606963	0,2606963
V41	~~	V10	19,291308	-0,1874826	-0,1874826	-0,524629	-0,524629
V15	~~	V29	18,727898	0,1729423	0,1729423	0,2638852	0,2638852
Symptoms	=~	V31	18,236825	-0,2616651	-0,2326925	-0,2326925	-0,2326925
Intensity	=~	V2	18,221073	-0,2696037	-0,248094	-0,248094	-0,248094
Symptoms	=~	V28	18,096215	0,3116467	0,27714	0,27714	0,27714
Prof_Dev	=~	V20	18,012831	-0,3109417	-0,1800624	-0,1800624	-0,1800624
Intensity	=~	V6	18,009677	-0,2227883	-0,2050137	-0,2050137	-0,2050137
V6	~~	V24	17,582198	-0,1731691	-0,1731691	-0,3390762	-0,3390762
V15	~~	V25	17,480705	0,1723339	0,1723339	0,4378127	0,4378127
V9	~~	V15	17,458251	0,1684678	0,1684678	0,2636189	0,2636189
Subjective_WB	=~	V28	17,092476	0,3101632	0,1936248	0,1936248	0,1936248
V9	~~	V14	16,806162	0,1776063	0,1776063	0,266986	0,266986
Harassment	=~	V1	16,645996	-0,3390797	-0,2293504	-0,2293504	-0,2293504
Health_Risk	=~	V1	16,241194	0,2306077	0,1837769	0,1837769	0,1837769
Intensity	=~	V40	16,049383	-0,1916431	-0,1763533	-0,1763533	-0,1763533

V43	~~	V12	15,730252	0,1411435	0,1411435	0,3340992	0,3340992
Symptoms	=~	V43	15,255616	-0,2708635	-0,2408725	-0,2408725	-0,2408725
Harassment	=~	V12	14,941813	-0,3183334	-0,2153178	-0,2153178	-0,2153178
Prof_Dev	=~	V43	14,589618	-0,2678066	-0,1550834	-0,1550834	-0,1550834
V4	~~	V9	14,581965	-0,1836898	-0,1836898	-0,2853672	-0,2853672
Intensity	=~	V31	14,392679	0,2544899	0,2341861	0,2341861	0,2341861
Symptoms	=~	V13	14,253702	-0,2585454	-0,2299183	-0,2299183	-0,2299183
V12	~~	V29	14,221517	-0,1795883	-0,1795883	-0,2803518	-0,2803518
V12	~~	V36	13,967626	-0,2691919	-0,2691919	-0,5297213	-0,5297213
Autonomy	=~	V18	13,940818	0,2200413	0,1903248	0,1903248	0,1903248
Intensity	=~	V15	13,79483	0,245112	0,2255563	0,2255563	0,2255563
Harassment	=~	V40	13,729096	0,2802812	0,1895796	0,1895796	0,1895796
Health_Risk	=~	V2	13,673891	-0,2097347	-0,1671427	-0,1671427	-0,1671427
V1	~~	V18	13,531455	-0,1790722	-0,1790722	-0,3441188	-0,3441188
V13	~~	V12	13,465043	0,1315397	0,1315397	0,2719425	0,2719425
Subjective_WB	=~	V15	13,415374	-0,3633934	-0,2268547	-0,2268547	-0,2268547
V20	~~	V40	13,28184	-0,1339246	-0,1339246	-0,423184	-0,423184
Subjective_WB	=~	V23	13,124206	-0,3423233	-0,2137013	-0,2137013	-0,2137013
Autonomy	=~	V41	13,056287	-0,1806391	-0,1562438	-0,1562438	-0,1562438
V43	~~	V8	12,793876	0,1421675	0,1421675	0,3236842	0,3236842
V12	~~	V30	12,701433	0,2409773	0,2409773	0,385555	0,385555
V17	~~	V30	12,582593	0,2402733	0,2402733	0,376308	0,376308
V41	~~	V27	12,489383	0,1818565	0,1818565	0,4408639	0,4408639
Social_Inclusion	=~	V23	12,474408	-0,3570909	-0,2477623	-0,2477623	-0,2477623
Harassment	=~	V8	12,457483	-0,3017227	-0,2040825	-0,2040825	-0,2040825
Harassment	=~	V2	12,447287	0,2951798	0,1996569	0,1996569	0,1996569
V6	~~	V43	12,307415	-0,1560387	-0,1560387	-0,3624677	-0,3624677
V8	~~	V12	11,981434	0,1479141	0,1479141	0,2420664	0,2420664
Autonomy	=~	V43	11,81782	0,1701894	0,1472054	0,1472054	0,1472054
V23	~~	V24	11,809938	-0,1370692	-0,1370692	-0,5356802	-0,5356802
V39	~~	V1	11,798977	0,1216355	0,1216355	0,3141968	0,3141968
V39	~~	V40	11,636082	0,08307	0,08307	0,2703375	0,2703375
V7	~~	V40	11,604285	-0,13724	-0,13724	-0,3492712	-0,3492712
V7	~~	V41	11,46564	-0,1422376	-0,1422376	-0,3991038	-0,3991038
V20	~~	V43	11,302972	-0,1216813	-0,1216813	-0,3988321	-0,3988321

V15	~~	V16	11,272244	0,1283818	0,1283818	0,2219341	0,2219341
V10	~~	V28	11,265894	-0,1536552	-0,1536552	-0,2755171	-0,2755171
Subjective_WB	=~	V18	11,218189	-0,340073	-0,2122965	-0,2122965	-0,2122965
V42	~~	V10	11,214019	-0,1570662	-0,1570662	-0,3560788	-0,3560788
V1	~~	V29	10,912135	-0,1750999	-0,1750999	-0,2909972	-0,2909972
Symptoms	=~	V24	10,905666	-0,2248023	-0,1999114	-0,1999114	-0,1999114
V7	~~	V43	10,768003	-0,1337693	-0,1337693	-0,3531312	-0,3531312
V7	~~	V24	10,738947	-0,1223987	-0,1223987	-0,2723619	-0,2723619
Health_Risk	=~	V42	10,629649	0,1599937	0,1275029	0,1275029	0,1275029
Harassment	=~	V18	10,418612	0,2735146	0,1850028	0,1850028	0,1850028
Subjective_WB	=~	V8	10,201936	0,3252121	0,2030194	0,2030194	0,2030194
V12	~~	V15	10,095706	-0,1416316	-0,1416316	-0,2355413	-0,2355413

Note. MI = modification index; EPC = expected parameter change; =~ indicates a potential cross-loading, whereas ~~ indicates a potential residual covariance. Only the principal modification indices are presented.

upplementary Table S4. Item-level factor loadings for the final eight-factor MPWES model in the validation sample (N = 1,231).

Factor	Item	Unstandardized estimate (B)	SE	p	Standardized loading (λ)
Subjective well-being	V6	1.000	—	—	0.617
Subjective well-being	V7	1.141	0.037	< 0.001	0.704
Subjective well-being	V13	1.215	0.040	< 0.001	0.749
Subjective well-being	V20	1.221	0.040	< 0.001	0.753
Subjective well-being	V39	1.342	0.043	< 0.001	0.828
Subjective well-being	V40	1.333	0.043	< 0.001	0.822
Subjective well-being	V41	1.420	0.045	< 0.001	0.876
Subjective well-being	V42	1.263	0.041	< 0.001	0.779
Subjective well-being	V43	1.259	0.040	< 0.001	0.776
Social inclusion	V1	1.000	—	—	0.662
Social inclusion	V2	0.883	0.037	< 0.001	0.584
Social inclusion	V4	0.953	0.038	< 0.001	0.631

Factor	Item	Unstandardized estimate (B)	SE	p	Standardized loading (λ)
Social inclusion	V8	0.876	0.035	< 0.001	0.580
Social inclusion	V10	0.996	0.036	< 0.001	0.659
Social inclusion	V17	1.025	0.039	< 0.001	0.678
Social inclusion	V9	0.904	0.040	< 0.001	0.598
Social inclusion	V12	0.944	0.040	< 0.001	0.625
Social inclusion	V14	1.016	0.040	< 0.001	0.672
Social inclusion	V15	0.878	0.039	< 0.001	0.581
Social inclusion	V16	1.110	0.038	< 0.001	0.734
Social inclusion	V18	1.114	0.041	< 0.001	0.738
Social inclusion	V29	0.801	0.040	< 0.001	0.530
Social inclusion	V3	0.908	0.039	<0.001	0.545
Social inclusion	V19	0.987	0.009	<0.001	0.654
Workplace harassment	V34	1.000	—	—	0.740
Workplace harassment	V35	1.070	0.092	< 0.001	0.792
Workplace harassment	V36	1.175	0.093	< 0.001	0.870
Workplace harassment	V33	1.210	0.891	<0.001	0.789
Work intensity	V23	1.000	—	—	0.954
Work intensity	V24	0.784	0.027	< 0.001	0.748
Work intensity	V27	0.426	0.032	< 0.001	0.407
Work intensity	V28	0.403	0.033	< 0.001	0.385
Psychosomatic symptoms	V37	1.000	—	—	0.886
Psychosomatic symptoms	V38	0.832	0.051	< 0.001	0.737

Factor	Item	Unstandardized estimate (B)	SE	p	Standardized loading (λ)
Professional development	V30	1.000	—	—	0.679
Professional development	V31	1.408	0.076	< 0.001	0.955
Professional development	V32	1.399	0.075	< 0.001	0.949
Health risks	V21	1.000	—	—	0.788
Health risks	V22	1.204	0.087	< 0.001	0.948
Autonomy	V25	1.000	—	—	0.873
Autonomy	V26	0.895	0.068	< 0.001	0.781

Note. The first item of each factor was used as the reference indicator, and its unstandardized loading was fixed to 1.000; therefore, no standard error or p-value was estimated for these items. SE = standard error. All freely estimated factor loadings were statistically significant at $p < 0.001$.

Table S5. Latent factor correlations with standard errors, z statistics, p-values, and 95% confidence intervals in the calibration and validation samples.

Sample	Factor_1	Factor_2	r	SE	z	p	CI_95_lower	CI_95_upper
Calibration (N = 400)	Harassment	Autonomy	0,148103422	0,08780073	1,686813101	0,091639296	-0,02398285	0,32018969
Calibration (N = 400)	Harassment	Health_Risks	-0,40861352	0,082898447	-4,92908529	8,26155E-07	-0,57109149	-0,24613555
Calibration (N = 400)	Harassment	Professional_Development	-0,11459286	0,093276848	-1,22852416	0,219250266	-0,29741212	0,068226401
Calibration (N = 400)	Harassment	Psychosomatic_Symptoms	0,541221528	0,084180343	6,429310097	1,28184E-10	0,376231088	0,706211968
Calibration (N = 400)	Harassment	Work_Intensity	-0,44785911	0,077770433	-5,7587324	8,47479E-09	-0,60028636	-0,29543186
Calibration (N = 400)	Health_Risks	Autonomy	-0,26785557	0,054619268	-4,9040491	9,3881E-07	-0,37490737	-0,16080378
Calibration (N = 400)	Professional_Development	Autonomy	-0,26259665	0,065131126	-4,03181495	5,53478E-05	-0,39025131	-0,13494199
Calibration (N = 400)	Professional_Development	Health_Risks	0,357575039	0,06873904	5,201920779	1,97239E-07	0,222848997	0,492301081
Calibration (N = 400)	Psychosomatic_Symptoms	Autonomy	0,030919115	0,077514018	0,398884177	0,689978548	-0,12100557	0,182843798
Calibration (N = 400)	Psychosomatic_Symptoms	Health_Risks	-0,17775456	0,076578493	-2,32120735	0,020275655	-0,32784565	-0,02766347
Calibration (N = 400)	Psychosomatic_Symptoms	Professional_Development	-0,09497027	0,086152727	-1,10234782	0,270310486	-0,26382651	0,073885971
Calibration (N = 400)	Social_Inclusion	Autonomy	0,499653835	0,041751883	11,9672168	0	0,417821648	0,581486022
Calibration (N = 400)	Social_Inclusion	Harassment	0,39509811	0,057534009	6,867209845	6,54699E-12	0,282333525	0,507862695
Calibration (N = 400)	Social_Inclusion	Health_Risks	-0,39149854	0,046228215	-8,46882241	0	-0,48210418	-0,30089291
Calibration (N = 400)	Social_Inclusion	Professional_Development	-0,51191287	0,051560098	-9,92846967	0	-0,61296881	-0,41085694
Calibration (N = 400)	Social_Inclusion	Psychosomatic_Symptoms	0,424006512	0,060355636	7,025135347	2,13851E-12	0,305711639	0,542301385
Calibration (N = 400)	Social_Inclusion	Work_Intensity	-0,67820615	0,032049357	-21,161303	0	-0,74102174	-0,61539057
Calibration (N = 400)	Subjective_WB	Autonomy	0,356613114	0,04603781	7,746092045	9,54792E-15	0,266380664	0,446845563

Calibration (N = 400)	Subjective_WB	Harassment	0,276512314	0,064236944	4,304568317	1,67312E-05	0,150610217	0,402414411
Calibration (N = 400)	Subjective_WB	Health_Risks	-0,24042952	0,048866182	-4,92016184	8,64727E-07	-0,33620548	-0,14465357
Calibration (N = 400)	Subjective_WB	Professional_Development	-0,31060201	0,05428042	-5,72217404	1,05169E-08	-0,41698968	-0,20421434
Calibration (N = 400)	Subjective_WB	Psychosomatic_Symptoms	0,634951151	0,047546082	13,35443689	0	0,541762542	0,728139759
Calibration (N = 400)	Subjective_WB	Social_Inclusion	0,802168585	0,019482473	41,17385861	0	0,76398364	0,840353531
Calibration (N = 400)	Subjective_WB	Work_Intensity	-0,64918912	0,031566413	-20,5658186	0	-0,71105816	-0,58732009
Calibration (N = 400)	Work_Intensity	Autonomy	-0,30514036	0,054951727	-5,55288019	2,81001E-08	-0,41284377	-0,19743695
Calibration (N = 400)	Work_Intensity	Health_Risks	0,552655381	0,048650818	11,35963172	0	0,457301529	0,648009232
Calibration (N = 400)	Work_Intensity	Professional_Development	0,396907175	0,061061663	6,500104192	8,02645E-11	0,277228514	0,516585836
Calibration (N = 400)	Work_Intensity	Psychosomatic_Symptoms	-0,61765374	0,053288989	-11,5906447	0	-0,72209824	-0,51320924
Validation (N = 1231)	Harassment	Autonomy	0,33959916	0,050320972	6,748660532	1,49216E-11	0,240971867	0,438226454
Validation (N = 1231)	Harassment	Health_Risks	-0,38840841	0,047670143	-8,14783385	4,44089E-16	-0,48184017	-0,29497664
Validation (N = 1231)	Harassment	Professional_Development	0,152001214	0,057595841	2,639100519	0,008312633	0,03911544	0,264886987
Validation (N = 1231)	Harassment	Psychosomatic_Symptoms	0,577255849	0,053963212	10,69721076	0	0,471489898	0,683021801
Validation (N = 1231)	Harassment	Work_Intensity	-0,64379141	0,046673022	-13,7936518	0	-0,73526886	-0,55231397
Validation (N = 1231)	Health_Risks	Autonomy	-0,34697985	0,0306599	-11,3170576	0	-0,40707215	-0,28688755
Validation (N = 1231)	Professional_Development	Autonomy	-0,10873496	0,04021181	-2,70405527	0,00684989	-0,18754866	-0,02992126
Validation (N = 1231)	Professional_Development	Health_Risks	-0,08289487	0,039144657	-2,11765478	0,034204315	-0,15961699	-0,00617275
Validation (N = 1231)	Psychosomatic_Symptoms	Autonomy	0,027947459	0,046452743	0,601632063	0,547419081	-0,06309824	0,118993162
Validation (N = 1231)	Psychosomatic_Symptoms	Health_Risks	-0,09097446	0,042581495	-2,13647878	0,032640409	-0,17443266	-0,00751626
Validation (N = 1231)	Psychosomatic_Symptoms	Professional_Development	0,075270559	0,050922134	1,478150136	0,139367597	-0,02453499	0,175076107
Validation (N = 1231)	Social_Inclusion	Autonomy	0,350625282	0,027292427	12,84698084	0	0,297133109	0,404117456
Validation (N = 1231)	Social_Inclusion	Harassment	0,584132171	0,032009063	18,24896192	0	0,521395561	0,646868782
Validation (N = 1231)	Social_Inclusion	Health_Risks	-0,19933776	0,030686775	-6,49588497	8,25466E-11	-0,25948274	-0,13919279
Validation (N = 1231)	Social_Inclusion	Professional_Development	-0,20875015	0,03476905	-6,00390715	1,92625E-09	-0,27689623	-0,14060406
Validation (N = 1231)	Social_Inclusion	Psychosomatic_Symptoms	0,429121945	0,03743505	11,46310587	0	0,355750595	0,502493296
Validation (N = 1231)	Social_Inclusion	Work_Intensity	-0,46900308	0,027151733	-17,2734126	0	-0,5222195	-0,41578666
Validation (N = 1231)	Subjective_WB	Autonomy	0,203268307	0,027682353	7,342883881	2,08944E-13	0,149011891	0,257524722
Validation (N = 1231)	Subjective_WB	Harassment	0,345928299	0,043557236	7,941924957	1,9984E-15	0,260557686	0,431298913
Validation (N = 1231)	Subjective_WB	Health_Risks	-0,09675067	0,030212074	-3,20238427	0,001362951	-0,15596525	-0,03753609
Validation (N = 1231)	Subjective_WB	Professional_Development	-0,13532059	0,0354565	-3,81652411	0,000135345	-0,20481405	-0,06582712
Validation (N = 1231)	Subjective_WB	Psychosomatic_Symptoms	0,651015495	0,029306378	22,21412344	0	0,59357605	0,70845494
Validation (N = 1231)	Subjective_WB	Social_Inclusion	0,684494274	0,01660039	41,23362602	0	0,651958107	0,71703044
Validation (N = 1231)	Subjective_WB	Work_Intensity	-0,54010908	0,023264101	-23,2164172	0	-0,58570588	-0,49451228
Validation (N = 1231)	Work_Intensity	Autonomy	-0,19577728	0,032896298	-5,95134679	2,65945E-09	-0,26025283	-0,13130172

Validation (N = 1231)	Work_Intensity	Health_Risks	0,431574654	0,029217772	14,77096403	0	0,374308874	0,488840434
Validation (N = 1231)	Work_Intensity	Professional_Development	-0,01032519	0,040733145	-0,25348369	0,799894461	-0,09016069	0,06951031
Validation (N = 1231)	Work_Intensity	Psychosomatic_Symptoms	-0,61020777	0,038077939	-16,0252308	0	-0,68483916	-0,53557638
